# AICO: Feature significance tests for supervised learning

**DOI:** 10.1073/pnas.2530045123

**Published:** 2026-09-23

**Authors:** Kay Giesecke, Enguerrand Horel, Chartsiri Jirachotkulthorn

**Affiliations:** ^a^ Hasso Plattner Institute, University of Potsdam, Potsdam 14482, Germany; ^b^Upstart Inc., San Mateo, CA 94402; ^c^ Institute for Computational and Mathematical Engineering, Stanford University, Stanford, CA 94305

**Keywords:** machine learning, inference, *P*-value, confidence interval, feature significance

## Abstract

Machine learning is central to modern science, industry, and policy, yet its predictive power often comes at the cost of transparency: We rarely know which input features drive a model’s predictions. Without such understanding, researchers cannot draw reliable conclusions, practitioners cannot ensure fairness or accountability, and policymakers cannot trust or govern model-based decisions. Existing tools for assessing feature influence are limited; most lack statistical guarantees, and many require costly retraining or surrogate modeling, making them impractical for large modern models. We introduce AICO (Add-In COvariates), a broadly applicable framework that turns model interpretability into an efficient statistical exercise. AICO tests whether each feature contributes to predictive performance by masking its information and measuring the resulting change. The method provides exact, finite-sample feature P-values and CIs for feature importance through a simple, nonasymptotic hypothesis testing procedure. It requires no retraining, surrogate modeling, or distributional assumptions, making it feasible for large-scale algorithms. In both controlled experiments and real applications, from credit scoring to mortgage-behavior prediction, AICO identifies variables that contribute to model behavior, providing a scalable and statistically principled path toward transparent and trustworthy machine learning.

## Significance

Machine-learning models now inform scientific discovery, industrial decision-making, and public policy, yet their inner workings often remain opaque. Understanding which features drive a model’s predictions is essential for transparency and trust. AICO (Add-In COvariates) provides a rigorous and efficient hypothesis-testing framework for quantifying feature importance in any predictive model. It delivers exact statistical inference in the form of feature P-values and CIs without retraining or auxiliary modeling, making transparent analysis feasible at modern scale. By enabling reliable identification of the variables that drive model behavior, AICO supports trustworthy, interpretable, and accountable machine learning across scientific, industrial, and policy domains.

Machine learning has become integral to modern science, industry, and public policy. Algorithms now predict molecular activity, identify disease markers, forecast financial defaults, and guide the allocation of social resources. Yet even as these systems achieve extraordinary predictive accuracy, their inner workings remain largely opaque. We often do not know which input features truly shape a model’s predictions, which contribute little to predictive performance, or which may encode unwanted biases. This opacity limits scientific understanding, hinders regulatory oversight, and undermines public trust in data-driven decision-making. Despite extensive progress in explainable AI, most tools for measuring feature importance lack statistical guarantees or depend on computationally costly retraining or surrogate modeling procedures, restricting their use in today’s large, complex models. As a result, reconciling interpretability with scalable inference remains an open challenge for modern machine-learning practice.

We introduce AICO (Add-In COvariates), a tractable method for feature significance testing in any supervised learning model, whether for regression or classification. AICO transforms the question of interpretability into one of formal statistical inference: It tests whether a feature contributes meaningfully to a model’s predictive performance, using only the model’s fitted predictions and observed outcomes. AICO constructs test statistics based on the change in model performance when a feature is masked. It is model- and distribution-agnostic: It makes no assumptions about the model class, data distribution, or estimand (for example, conditional mean or quantile). The method does not rely on retraining or surrogate models, making it well suited for analyzing complex, high-dimensional algorithms that are costly to train. It bridges a gap between machine-learning practice and classical statistical inference, offering a tractable route to transparent, trustworthy prediction in today’s large-scale applications.^*^

The AICO feature effect—the change in model performance due to masking—quantifies a feature’s importance for each individual sample. For a null feature, these effects form a distribution across test samples whose median is nonpositive. A uniformly most powerful randomized sign test achieves finite-sample exactness: Its size (type-I error probability) exactly matches the nominal level (e.g., 5%). The procedure produces exact, randomized feature P-values whose conditional distribution, given the data, is uniform on an interval that can be reported in practice. It is simple, robust, and computationally efficient, relying on minimal assumptions and remaining valid under broad conditions. These properties enable valid inference on a model’s features without relying on large-sample approximations or parametric assumptions.

The median feature effect serves as a natural, robust, and global measure of feature importance. The choice of masking specification gives rise to alternative importance scores: A conditional score measures the incremental contribution of a feature given the others, whereas an unconditional score quantifies its stand-alone contribution. We develop randomized, exact-coverage CIs for the median that are dual to the hypothesis test. Together with the test and corresponding P-values, these intervals provide a unified framework for feature inference in any learning model.

Extensive experiments on synthetic “known-truth” tasks demonstrate the statistical and computational performance of our approach in neural-network regression and classification settings. We compare AICO with powerful, model-agnostic, and nonasymptotic tests by refs. 1–3, as well as with the model-X knockoff procedures of refs. 4 and 5 for variable selection under false discovery control. Our test identifies relevant features comparably to these alternatives while producing fewer discoveries among features that are null in the data-generating process, all while reducing computational cost by orders of magnitude. The test and median feature-importance measure remain stable across a range of feature distributions, including settings with strong correlations and interactions. The median is robust to noise, producing stable feature rankings.

Empirical analyses demonstrate the ability of our framework to inform high-stakes decisions with legal, economic, and regulatory implications. In a credit scoring task using ensembles of gradient-boosting models, AICO identifies several statistically significant features describing past payment behavior and social factors. In a mortgage state–transition prediction task based on a recently proposed set–sequence model (6) and trained on extensive panel data, AICO detects time-varying macroeconomic variables such as mortgage rates, as well as features summarizing past state transitions, as significant predictors of borrower behavior.

## 1.1. Related literature.

There is a large body of work developing algorithmic feature-importance measures; see ref. 7 for an overview and ref. 8 for a unifying framework for interpretability methods. Popular examples are based on Shapley values [e.g., SHAP (9)], local approximations [e.g., LIME (10)], and permutation methods (e.g., refs. 11 and 12). These methods provide useful insight into feature contribution but typically lack formal statistical guarantees and associated P-values, in contrast to the importance measures delivered by our hypothesis-testing framework. Moreover, algorithmic importance measures can be sensitive to feature correlation (13, 14), whereas the conditional AICO score is robust to both correlation and interaction. Finally, we show that AICO achieves significant improvements in computational efficiency relative to widely used algorithmic approaches.

The statistical and econometric literature has long studied significance testing for variables in nonparametric regression models. (15–19) and others develop kernel-based asymptotic testing procedures. (20–22) and others develop gradient-based asymptotic tests using neural networks. Coleman et al. (23) develop permutation-based asymptotic tests for random forests; see also Mentch and Hooker (24) for an alternative approach based on U-statistics. In contrast, AICO provides a model-agnostic, nonasymptotic framework that delivers exact P-values and CIs and applies to a broad range of data structures.

Pursuing a model-agnostic approach, refs. 1, 2, 4, and 25–27 and others develop nonasymptotic tests for conditional independence in the model-X knockoff framework of ref. 4. Refs. 28–31 and others study asymptotic tests for conditional mean independence. These procedures assess feature significance through the change in predictive performance when a feature is removed from the model. Related feature-importance measures based on this principle are studied—often via asymptotic analysis—in refs. 3 and 30–34.

AICO studies an alternative formulation of this incremental-value principle based on feature masking rather than feature removal, while delivering exact, nonasymptotic inferential guarantees. This masking strategy avoids the repeated model retraining or auxiliary modeling required by existing approaches.^†^ As a result, AICO achieves substantial gains in computational efficiency and enables inference for large, feature-rich models that are otherwise computationally challenging. Our experiments show that these computational gains do not come at the expense of statistical performance.

Like several of the aforementioned methods, AICO employs data splitting into training and testing samples to facilitate inference after model selection; see ref. 34 and the references therein. While data splitting can reduce statistical efficiency, it is widely used in machine learning to evaluate the out-of-sample performance of fitted models, and our approach aligns with this standard practice. Moreover, a power analysis allows the training and testing sample sizes to be chosen to achieve a desired level of power. Even under unfavorable alternatives, the test sample sizes required for high power remain small relative to typical data sizes in machine learning. Finally, the AICO framework admits a cross-fitting implementation, which attains comparable performance while eliminating reliance on a single data split.

## 2. Feature Effect

We consider a distribution-free setting, where the random pair Z=(X,Y) of d-dimensional feature $X=(X^{1},\ldots ,X^{d})\in X$ and response Y∈Y has an unknown distribution. In a typical regression formulation with tabular data, $X\subseteq R^{d}$ and Y=R. We observe data $Z_{1},\ldots ,Z_{m}$, where the $Z_{i}=\left(X_{i},Y_{i}\right)\in Z=X\times Y$ are independent copies of Z.

We assume a random split of the index set [m]={1,…,m} into nonintersecting subsets $I_{1}$ and $I_{2}$. The dataset $D_{1}={\left\{Z_{i}\right\}}_{i\in I_{1}}$ is used for training, while $D_{2}={\left\{Z_{i}\right\}}_{i\in I_{2}}$ is reserved for testing. Such sample splitting is standard in machine learning and, in our setting, enables valid postselection inference.^‡^ The results below provide guidance on selecting the size of $I_{2}$ to optimize testing power. As discussed in *SI Appendix*, section S3, our approach naturally extends to cross-fitting with multiple data splits, allowing each sample $Z_{i}$ to be used for both training and testing while maintaining valid inference.

We also take as given a model (algorithm) f:X→M trained on $D_{1}$, which is to be evaluated. Throughout, we treat f as a nonrandom function of $D_{1}$. We impose no restrictions on the form of the estimand: f may represent the conditional mean, a quantile, or any other statistic of the response. Nor do we assume a particular training criterion or optimization procedure for fitting f.

We test the statistical significance of a feature ℓ∈[d] under the fitted f. When f is linear, classical asymptotic procedures such as the t test are available. In contrast, we develop a simple, nonasymptotic, and model-agnostic procedure that does not depend on a particular model structure and delivers valid inference for f of arbitrary form. As is standard in postselection inference with data splitting, testing based on $D_{2}$ is conducted conditional on the training data $D_{1}$ and on the training and selection procedures used to construct f (e.g., ref. 34).

### 2.1. Main Ideas.

We sketch our approach in a standard regression setting in which f(x) estimates the conditional mean E(Y|X=x) of a continuous, real-valued response Y.^§^ Section 2.2 extends the framework to other types of responses and estimands. Following a growing literature and prediction-focused downstream applications of machine-learning models, our approach ties the statistical significance of a feature to its incremental predictive power (3, 28–31, 34). Rather than assessing this predictive contribution through feature removal and model retraining, we propose a masking-based strategy for measuring a feature’s incremental predictive value. We posit that if feature ℓ has predictive power, then masking its values systematically affects prediction errors. We develop a simple, nonasymptotic hypothesis testing procedure to detect this effect.

We first measure this effect at the level of an individual test sample $i\in I_{2}$ with feature–response pair $\left(X_{i},Y_{i}\right)$. Consider the masked feature vector

[1] $$\begin{array}{c} m^{\ell }\left(X_{i}\right)=\left(X_{i}^{1},\ldots ,v^{\ell },\ldots ,X_{i}^{d}\right), \end{array}$$

where the observed value $X_{i}^{\ell }$ of feature ℓ is replaced by a reference value $v^{\ell }\neq X_{i}^{\ell }$. A natural choice for $v^{\ell }$ is a measure of central tendency of ℓ in the training data $D_{1}$, such as the mean when ℓ has a continuous distribution on an interval. The model output $f(m^{\ell }\left(X_{i}\right))$ represents the prediction when the masked feature vector [**1**] is used as input. The prediction error can be quantified by ${\left\{f\left(m^{\ell }\left(X_{i}\right)\right)-Y_{i}\right\}}^{2}$, which is natural when f is trained using a squared-error criterion. The feature effect for sample i is the incremental error due to masking ℓ,

[2] $$\begin{array}{c} {\left\{f\left(m^{\ell }\left(X_{i}\right)\right)-Y_{i}\right\}}^{2}-{\left\{f\left(X_{i}\right)-Y_{i}\right\}}^{2}. \end{array}$$

The sign and magnitude of the feature effect [**2**] characterize the change in predictive accuracy induced by masking feature ℓ for sample i under f. If Eq. **2** is positive, masking ℓ reduces accuracy, indicating that ℓ contributes to the fitted model’s predictive performance for sample i. In this case, larger values correspond to greater reductions in accuracy due to masking and hence greater predictive power of ℓ. Thus, the magnitude of Eq. **2** quantifies the effect size of feature ℓ at the sample level.

Moving beyond an individual sample, we deem feature ℓ significant when masking it systematically degrades predictive accuracy across the test set. Consider the median of the sample-level feature effects [**2**] across $I_{2}$. A positive median is evidence that masking ℓ systematically reduces predictive accuracy and hence that ℓ is significant. Conversely, a nonpositive median is treated as absence of evidence that ℓ is significant. A sign test of this property delivers exact P-values for feature significance (Section 3). The median feature effect also serves as a point estimate of feature importance. Larger positive values correspond to greater reductions in predictive accuracy due to masking and hence greater predictive power of ℓ. CIs for feature importance are obtained by inverting the test (Section 4).

An analysis of incremental prediction errors also underlies significance testing based on conditional mean independence (3, 28–31, 34). This approach introduces a reduced feature vector $X^{-\ell }=\left(X^{1},\ldots ,X^{\ell -1},X^{\ell +1},\ldots ,X^{d}\right)$ that excludes $X^{\ell }$. Feature ℓ is treated as having no incremental predictive value if the response Y is conditionally mean independent of ℓ given $X^{-\ell }$, i.e., if

[3] $$\begin{array}{c} {\left\{E\left(Y|X^{-\ell }\right)-Y\right\}}^{2}-{\left\{E\left(Y|X\right)-Y\right\}}^{2} \end{array}$$

has mean zero. The quantity [**3**] measures the incremental squared prediction error induced by removing ℓ from the feature set. Our feature effect [**2**] is based on the same incremental prediction-error principle, but differs from Eq. **3** in two important respects.^¶^ First, Eq. **2** measures the change in prediction error induced by masking ℓ rather than removing it, thereby avoiding the need to construct an auxiliary model for $E(Y|X^{-\ell })$. This yields substantial computational gains, especially when many features are analyzed or f is large. It also avoids the additional randomness introduced by auxiliary model fitting. Second, our approach focuses on sample-level prediction errors under masked features, rather than population-level errors. This sample-level formulation naturally supports an assumption-light, nonasymptotic framework for significance testing.

### 2.2. General Formulation.

We formulate our approach in greater generality, allowing for real and categorical responses Y and estimands beyond the conditional mean such as conditional quantiles. Recall our premise: If feature ℓ is predictive, then masking its true values will systematically impact prediction errors under f. The feature effect measures this impact at the sample level; its distribution across the test set will be used to construct test statistics.

We begin by specifying a masking function $m^{\ell }:X\to X$ that masks the ℓth and possibly other components of an observed feature vector $X_{i}$ with suitable reference values, and an unmasking function $u^{\ell }:X\to X$ that restores some or all of the true feature values. The reference values are allowed to depend on the training data $D_{1}$, in which case $m^{\ell }$ and $u^{\ell }$ are random functions. The errors associated with the predictions $f(m^{\ell }\left(X_{i}\right))$ and $f(u^{\ell }\left(X_{i}\right))$ for masked and unmasked feature vectors, respectively, will then be used to define the feature effect for sample i. Before doing that, we provide two canonical examples for the masking and unmasking functions and discuss the choice of the reference values. The first example reflects the formulation in Section 2.1 above.

Example 2.1: For all $x=(x^{1},\ldots ,x^{d})\in X$, let

[4] $$\begin{array}{c} m^{\ell }\left(x\right)=\left(x^{1},\ldots ,v^{\ell },\ldots ,x^{d}\right),\;u^{\ell }\left(x\right)=x. \end{array}$$

Feature ℓ is masked with a reference value $v^{\ell }$ so that the prediction $f(m^{\ell }\left(X_{i}\right))$ is based on $v^{\ell }$ rather than the true value $X_{i}^{\ell }$. To unmask, the true value is added back in—an operation that gives rise to the name AICO (Add-In COvariates). Since masking affects only ℓ, Eq. **4** can be used to construct a conditional feature effect, which measures the incremental contribution of ℓ given the others.

Example 2.2: For all x∈X, let

[5] $$\begin{array}{c} m^{\ell }\left(x\right)=\left(v^{1},\ldots ,v^{d}\right),\;u^{\ell }\left(x\right)=\left(v^{1},\ldots ,x^{\ell },\ldots ,v^{d}\right). \end{array}$$

Each feature j∈[d] is masked with a reference value $v^{j}$. Adding the true value of feature ℓ back into this context performs the same AICO operation. By masking all features, Eq. **5** can be used to construct an unconditional feature effect, which measures ℓ’s stand-alone contribution.

The masking and unmasking functions in Eqs. **4** and **5** require the specification of reference values $v^{j}$. These values should provide a neutral baseline for feature-effect measurement while mapping masked inputs to regions of the feature space where the model can be expected to provide reliable predictions. This suggests selecting suitable measures of central tendency from the observed values of each feature in the training set $I_{1}$, ensuring that masked feature values remain in the feature’s support.^#^ Specifically, if feature j has a continuous distribution with interval support in R, a natural choice for $v^{j}$ is the mean of ${\left\{X_{k}^{j}\right\}}_{k\in I_{1}}$. If feature j has a discrete distribution with support set $s^{j}$, such as a categorical feature, a natural choice is the adjusted mode $\arg\;\max_{w\in s^{j}\setminus \left\{x^{j}\right\}}p^{j}\left(w\right)$, where $x^{j}$ is the feature value being masked and $p^{j}$ denotes the probability mass function of ${\left\{X_{k}^{j}\right\}}_{k\in I_{1}}$. For example, if j is a dummy variable, then $v^{j}=1-x^{j}$. The adjusted mode generalizes naturally to categorical variables encoded by multiple dummies and ensures that the masked feature value differs from the original value while remaining in the feature’s support. Our numerical experiments in Section 5 and *SI Appendix*, section S2 highlight the effectiveness and robustness of these choices for $v^{j}$.

We construct the feature effect using masking and unmasking functions, generalizing [**2**]. Formally, the feature effect $\Delta^{\ell }\left(Z_{i}\right)$ is given by a random function $\Delta^{\ell }:Z\to R$ of a test sample $Z_{i}$, depending on the training data $D_{1}$. To allow for different types of response variables Y and estimands when specifying $\Delta^{\ell }$, we introduce a real-valued loss function L on M×Y that measures the discrepancy between the response predicted by the model f and the true response. A natural choice for L is the loss function used to train f, which ensures consistency across training and testing. Examples include the squared loss in regression used in Eq. **2**, the cross-entropy loss in classification (i.e., categorical Y), and the pinball loss in quantile regression.^‖^

Definition 2.1: The feature effect $\Delta^{\ell }\left(Z_{i}\right)$ is defined as the incremental loss for test sample $i\in I_{2}$ induced by masking feature ℓ. For z=(x,y)∈Z, it is given by

[6] $$\begin{array}{c} \Delta^{\ell }\left(z\right)=L\left(f\left(m^{\ell }\left(x\right)\right),y\right)-L\left(f\left(u^{\ell }\left(x\right)\right),y\right). \end{array}$$

The definition [**6**] can be paired with the masking functions in Examples 2.1 and 2.2. If Eq. **4** is used, $\Delta^{\ell }\left(Z_{i}\right)$ defines a conditional feature effect that measures the incremental loss due to masking ℓ given the true values of the other features and thus captures the conditional contribution of a feature. A special case is our initial specification [**2**], which arises from choosing the squared loss $L\left(b,y\right)={\left(b-y\right)}^{2}$. If Eq. **5** is used in Eq. **6**, the feature effect $\Delta^{\ell }\left(Z_{i}\right)$ measures the incremental loss due to masking ℓ while keeping the other features masked and thus captures the stand-alone contribution of a feature. We numerically evaluate both formulations in Section 5.

The feature-effect framework extends naturally along several dimensions. First, while our discussion has focused on a single feature ℓ∈[d], the same approach applies to subsets of [d], enabling joint testing of multiple features and the exploration of higher-order effects. Second, the framework accommodates panel data, where collections of units are observed over time; see *SI Appendix*, section S4. Third, it accommodates missing data; see *SI Appendix*, section S5.

## 3. Hypothesis Testing

To construct a hypothesis test for the significance of feature ℓ under the model f, we examine the empirical distribution of the feature effects $\Delta^{\ell }\left(Z_{i}\right)$ across the test samples $i\in I_{2}$. A positive center of this distribution is treated as evidence of significance, since this implies that a majority of the test samples have positive feature effects. Several classical tests could be used to assess such central tendencies, including t-, sign, Wilcoxon, and permutation tests. We propose a sign test for the median of the feature effects, which requires minimal assumptions on the data while retaining favorable asymptotic relative efficiency compared with a t test.

Assumption 3.1. *Conditional on the training data* $D_{1}$, *the feature effects* ${\left\{\Delta^{\ell }\left(Z_{i}\right)\right\}}_{i\in I_{2}}$ *are conditionally independent draws from a distribution* $P_{M^{\ell }}$ *with continuous distribution function* $F^{\ell }$ *and unique median* $M^{\ell }$.

We note that $P_{M^{\ell }}$ and $F^{\ell }$ in *Assumption* 3.1 represent the conditional distribution and distribution function of the feature effects given the training data $D_{1}$, and that $M^{\ell }$ denotes the corresponding conditional median. A sufficient condition for uniqueness of $M^{\ell }$ is that $F^{\ell }$ be strictly increasing in a neighborhood of $M^{\ell }$.

Under *Assumption* 3.1, the hypotheses for testing the significance of feature ℓ under f can be formulated in terms of $M^{\ell }$. For real $M_{0}$, consider

[7] $$\begin{array}{c} H_{0}:M^{\ell }\leq M_{0}\;\;\text{vs.}\;H_{1}:M^{\ell }>M_{0}. \end{array}$$

Take $M_{0}=0$. Under the null hypothesis, $M^{\ell }\leq 0$, so masking feature ℓ does not systematically degrade predictive accuracy across the test set. Under the alternative, $M^{\ell }>0$, so masking ℓ systematically increases prediction errors. While the choice $M_{0}=0$ is natural, Eq. **7** accommodates alternative values.

Let $B_{n,s}$ denote the Binomial distribution with parameters n∈N and s∈(0,1). Write $\Pi_{n,s}$ for its distribution function, $\pi_{n,s}$ for its probability function, and $q_{\lambda }\left(B_{n,s}\right)$ for its (lower) quantile at level λ∈(0,1). *Proposition* 3.2 below provides an optimal sign test for [**7**].

Proposition 3.2. *Under Assumption 3.1, a Uniformly Most Powerful test of size* α∈(0,1) *rejects* $H_{0}$ *with probability* $\phi_{N}\left(n_{+}^{\ell }\left(M_{0}\right),\alpha \right)$ *where* $n_{+}^{\ell }\left(w\right)=\#\left\{i\in I_{2}:\Delta^{\ell }\left(Z_{i}\right)>w\right\}$ *is the number of feature effect samples in the test set exceeding* w∈R and

[8] $$\begin{array}{c} \phi_{N}\left(n,\alpha \right)=1\left\{n>T_{N,\alpha }\right\}+\gamma_{N}\left(n,\alpha \right)1\left\{n=T_{N,\alpha }\right\}, \end{array}$$

where $N=|I_{2}|$ is the test sample size, $T_{N,\alpha }=q_{1-\alpha }\left(B_{N,1/2}\right)$ and

[9] $$\begin{array}{c} \gamma_{N}\left(n,\alpha \right)=\frac{\Pi_{N,1/2}\left(n\right)-\left(1-\alpha \right)}{\pi_{N,1/2}\left(n\right)},\;n=0,1,\ldots ,N. \end{array}$$

For brevity, we often suppress the dependence of the test statistic $n_{+}^{\ell }$ on $M_{0}$. The test rejects the null $H_{0}$ with probability one if $n_{+}^{\ell }$, the number of feature effects in the test set exceeding $M_{0}$, exceeds $T_{N,\alpha }$, the (1−α)-quantile of $B_{N,1/2}$. If $n_{+}^{\ell }$ lies exactly on the boundary $T_{N,\alpha }$ of the rejection region, the test randomizes, rejecting $H_{0}$ with probability $\gamma_{N}\left(n_{+}^{\ell },\alpha \right)$. Relative to a nonrandomized test of $H_{0}$ with test function $1\{\cdot >T_{N,\alpha }\}$, this randomization renders the procedure slightly more aggressive. Indeed, the randomized test achieves maximum power while guaranteeing *exactness*, in the sense that the size satisfies

[10] $$\begin{array}{c} \max_{M^{\ell }\leq M_{0}}E_{M^{\ell }}\left(\phi_{N}\left(n_{+}^{\ell },\alpha \right)\right)=\alpha , \end{array}$$

for any $M_{0}$ in [**7**], with the maximum attained at $M^{\ell }=M_{0}$. Here and below, $E_{M^{\ell }}$ denotes expectation under the feature effect sampling distribution $P_{M^{\ell }}$ of *Assumption* 3.1. Under $P_{M^{\ell }}$, the test statistic $n_{+}^{\ell }$ has a Binomial distribution $B_{N,s}$, where $s=1-F^{\ell }\left(M_{0}\right)=1/2$ when $M^{\ell }=M_{0}$ and s<1/2 when $M^{\ell }<M_{0}$.

The test statistic depends on the masking specification through the feature-effect construction [**6**]. The specification in Eq. **4** yields a conditional significance test tied to the incremental contribution of a feature given the others, whereas Eq. **5** yields a test based on its stand-alone contribution. Our numerical results in Section 5 and *SI Appendix*, section S2 suggest that the conditional test exhibits greater robustness in the presence of feature interactions and correlations.

In the context of the randomized test in Proposition 3.2 the notion of P-value is ambiguous. A conventional P-value could be defined as $\inf\{\alpha :\phi_{N}\left(n_{+}^{\ell },\alpha \right)=1\}$, the smallest level of significance α at which $H_{0}$ is rejected with probability one, which is $1-\Pi_{N,1/2}\left(n_{+}^{\ell }-1\right)$. (This is also a P-value for a nonrandomized test of $H_{0}$ with test function $1\{\cdot >T_{N,\alpha }\}$.) However, this formulation ignores the randomness of the test’s decision rule [**8**]. We follow ref. 37 and consider a randomized P-value $p(n_{+}^{\ell })$ with conditional distribution function given the test statistic specified by

[11] $$\begin{array}{c} P_{M^{\ell }}\left(p\left(n_{+}^{\ell }\right)\leq \alpha \;|\;n_{+}^{\ell }\right)=\phi_{N}\left(n_{+}^{\ell },\alpha \right),\;\alpha \in \left(0,1\right) \end{array}$$

for any $M^{\ell }\leq M_{0}$. From Eq. **8**, this function represents a uniform distribution on an interval $\left(a_{N}\left(n_{+}^{\ell }\right),b_{N}\left(n_{+}^{\ell }\right)\right)$ of length $\pi_{N,1/2}\left(n_{+}^{\ell }\right)$ where

$$\begin{array}{cc} a_{N}\left(n\right) & =1-\Pi_{N,1/2}\left(n\right),\\ b_{N}\left(n\right) & =1-\Pi_{N,1/2}\left(n-1\right), \end{array}$$

the right endpoint being the conventional P-value. By iterated expectations and Eq. **10**,

[12] $$\begin{array}{c} \max_{M^{\ell }\leq M_{0}}P_{M^{\ell }}\left(p\left(n_{+}^{\ell }\right)\leq \alpha \right)=\alpha . \end{array}$$

**Fig. 1.**
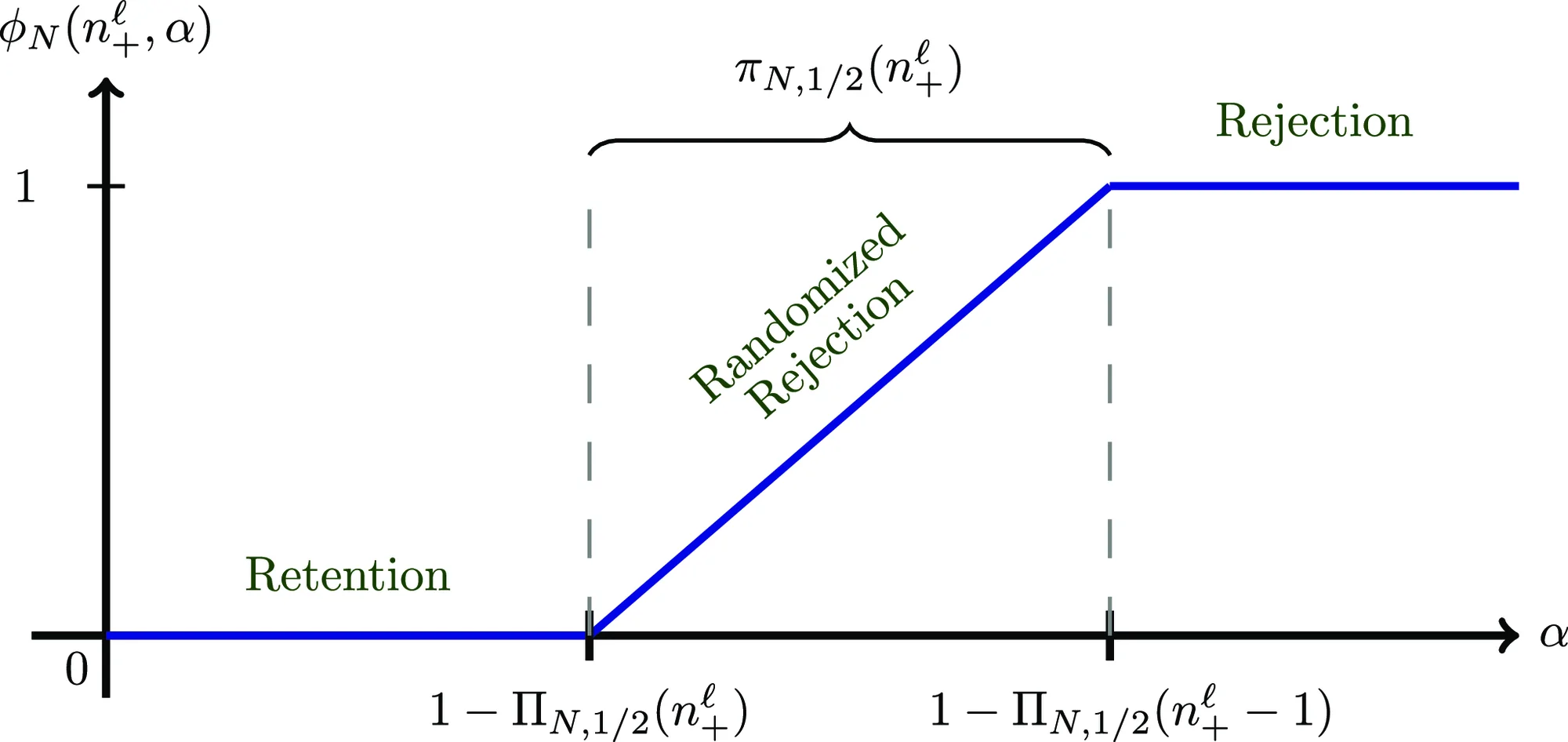
Distribution of randomized P-value and decision intervals.

Eq. **12** states that the randomized P-value $p(n_{+}^{\ell })$ is standard uniform under the “least favorable” sampling distribution, for which $M^{\ell }=M_{0}$. In this sense, $p(n_{+}^{\ell })$ is exact, mirroring the exactness of the test. If $p(n_{+}^{\ell })$ is concentrated below a given level α, or equivalently, if the conventional P-value $b_{N}\left(n_{+}^{\ell }\right)\leq \alpha$, we reject the null. If $p(n_{+}^{\ell })$ is concentrated above α, or equivalently, if $\alpha <a_{N}\left(n_{+}^{\ell }\right)$, we retain the null. Else the evidence is equivocal; we thus randomize by drawing a sample u from $U(a_{N}\left(n_{+}^{\ell }\right),b_{N}\left(n_{+}^{\ell }\right))$ and reject if u≤α. See Fig. 1 for a visualization. In practice, it suffices to report the interval $\left(a_{N}\left(n_{+}^{\ell }\right),b_{N}\left(n_{+}^{\ell }\right)\right)$ to fully characterize the P-value; there is often no need to force a decision.

The multiple testing problem can also be addressed in the randomized setting. A standard Bonferroni adjustment with test function $\phi_{N}\left(n,\alpha /d\right)$ and P-value being conditionally uniform on the interval $\left(da_{N}\left(n_{+}^{\ell }\right)\wedge 1,db_{N}\left(n_{+}^{\ell }\right)\wedge 1\right)$, where d is the dimension of the feature vector, controls the family-wise error rate (the probability of having at least one false discovery), see ref. 38. For a less conservative approach, one can also control the false discovery rate (39). Kulinskaya and Lewin (38) develop a formula and an algorithm for the adjusted rejection probability. Habiger (40) proposes an alternative Monte Carlo approach. Cousido-Rocha et al. (41) provide a comparison of methods.

The power function $H_{N,\alpha }\left(s\right)$ of the test is given by

$$\begin{array}{c} H_{N,\alpha }\left(s\right)=1-\Pi_{N,s}\left(T_{N,\alpha }\right)+\gamma_{N}\left(T_{N,\alpha },\alpha \right)\pi_{N,s}\left(T_{N,\alpha }\right), \end{array}$$

where $s=1-F^{\ell }\left(M_{0}\right)\in \left(1/2,1\right)$ is the parameter of the test statistic’s Binomial distribution $B_{N,s}$ under $H_{1}$. We can invert this function to determine the test set size N required to achieve a desired power level. Even when s is only slightly larger than 1/2, achieving power close to 1 requires N on the order of $10^{4}$, which remains modest relative to typical data sizes in machine learning. A pilot sample can be used to estimate s (42).

## 4. Measuring Feature Importance

We provide tractable measures of feature importance (effect size) that complement our significance test and the associated feature P-values. A local measure of importance is given by the feature effect $\Delta^{\ell }\left(Z_{i}\right)$, which captures the impact of a feature on predictive performance at the level of an individual sample $i\in I_{2}$. Larger feature effects indicate greater influence at the sample level.

At the global level, a natural measure of feature importance is the median $M^{\ell }$ of the conditional feature-effect distribution $F^{\ell }$ from which the $\Delta^{\ell }\left(Z_{i}\right)$ are drawn under *Assumption* 3.1. This median defines the feature significance hypotheses [**7**] being tested using the $\Delta^{\ell }\left(Z_{i}\right)$. When positive, $M^{\ell }$ measures the extent to which masking feature ℓ systematically degrades predictive accuracy across the test set. Larger positive values indicate a greater contribution of ℓ to predictive performance and hence greater importance.

The value of $M^{\ell }$ is unknown. A robust estimator is the empirical median of the feature effects across the test set,

[13] $$\begin{array}{c} {\hat{M}}^{\ell }=\mathrm{Median}\left\{\Delta^{\ell }\left(Z_{i}\right):i\in I_{2}\right\}. \end{array}$$

Under *Assumption* 3.1, this estimator is consistent and (approximately) equally likely to overestimate $M^{\ell }$ as to underestimate it. Moreover, it is unbiased when $F^{\ell }$ is symmetric about $M^{\ell }$; see section 4.4 of ref. 43. The test, P-value, and importance score [**13**] together provide a unified framework for feature inference in any model.

The choice of masking specification in the construction of the feature effect $\Delta^{\ell }\left(Z_{i}\right)$ in Eq. **6** gives rise to alternative importance scores. The specifications in Eqs. **4** and **5** induce conditional and unconditional scores, respectively. The conditional score measures the incremental contribution of a feature given the others, whereas the unconditional score quantifies its stand-alone contribution. Our numerical results in Section 5 and *SI Appendix*, section S2 indicate that the conditional score is more robust to feature interactions and correlations. This robustness represents an advantage over alternative importance measures such as SHAP (9), LOCO (34), and permutation importance (11, 12), which are sensitive to feature correlation (13, 14).

The resulting importance scores also depend on the choice of masking reference values. This dependence is natural and reflects the fact that predictive importance is inherently context-dependent. Accordingly, Eq. **13** quantifies the predictive importance of a feature relative to a specified masking scheme and study cohort. This interpretation is consistent with the broader feature-importance literature, where variable importance generally depends on the data distribution, prediction task, and feature perturbation mechanism.

The choice of loss function L in the feature-effect construction [**6**] can change the magnitude of the scores but generally preserves the feature rankings, indicating robustness (Section 5). The choice of L should therefore be guided by the aspect of prediction quality of interest in the application. In practice, setting L equal to the training loss function provides a natural default and ensures consistency across training and inference.

Since there is no ground truth for feature importance (44), multiple measures can be meaningful. Our score [**13**] offers an alternative to existing approaches. Relative to algorithmic measures such as SHAP and permutation-based scores, Eq. **13** has the additional advantage of being tied to a hypothesis test that also delivers feature P-values. Moreover, as shown below, the testing framework yields CIs for feature importance that algorithmic measures lack. Finally, as demonstrated in Section 5, our approach achieves orders-of-magnitude improvements in computational efficiency. Unlike existing methods, it can be used to measure feature importance even for the largest, feature-rich models.

### CIs.

To complement the point estimator [**13**], we construct CIs for $M^{\ell }$ by inverting the test [**8**]. Let $\Delta_{\left(1\right)}^{\ell }\leq \Delta_{\left(2\right)}^{\ell }\leq \cdots \leq \Delta_{\left(N\right)}^{\ell }$ denote the ordered sequence of the $\Delta^{\ell }\left(Z_{i}\right)$ across $I_{2}$. Define $\Delta_{\left(0\right)}^{\ell }=-\infty$ and $\Delta_{\left(N+1\right)}^{\ell }=\infty$.

Proposition 4.1. *Let* U *be a standard uniform variable that is independent of all other random elements including the data. Define a randomized CI for* $M^{\ell }$ *by*

[14] $$\begin{array}{c} C_{N}^{\ell }\left(\alpha \;;U\right)=\left\{\begin{array}{cc} {}[\Delta_{\left(N-T_{N,\alpha }\right)}^{\ell },\infty ), & 1-\gamma_{N}\left(T_{N,\alpha },\alpha \right)\geq U\\ {}[\Delta_{\left(N-T_{N,\alpha }+1\right)}^{\ell },\infty ), & \text{else}, \end{array}\right. \end{array}$$

*where* $T_{N,\alpha }$ *and* $\gamma_{N}\left(n,\alpha \right)$ *are as defined in Proposition 3.2. Under Assumption 3.1, the CI [**14**] has coverage probability* 1−α *for* α∈(0,1).

In analogy to the randomized P-value, the randomized CI [**14**] inherits the exactness of the test’s decision rule [**8**]. If $M_{0}<\Delta_{\left(N-T_{N,\alpha }\right)}^{\ell }$ we reject the null. If $\Delta_{\left(N-T_{N,\alpha }+1\right)}^{\ell }\leq M_{0}$ we retain the null. Else we reject with probability $\gamma_{N}\left(T_{N,\alpha },\alpha \right)$. In practice, it suffices to report the interval endpoints along with the rejection probability.

Standard arguments lead to alternative two-sided CIs, which are not, however, dual to the test [**8**]. Observe that the probability of $M^{\ell }\in \left[\Delta_{\left(k\right)}^{\ell },\Delta_{\left(k+1\right)}^{\ell }\right]$ equals $\pi_{N,1/2}\left(k\right)$. Using these probabilities, we can combine intervals of this form so as to attain a desired confidence coefficient. Choosing the intervals symmetrically, we obtain intervals

[15] $$\begin{array}{c} c_{m,N}^{\ell }=\left[\Delta_{\left(1+m\right)}^{\ell },\Delta_{\left(N-m\right)}^{\ell }\right] \end{array}$$

with coverage probability $1-2\Pi_{N,1/2}\left(m\right)$, where we select m∈N so that $\Pi_{N,1/2}\left(m\right)\leq \alpha /2$ for given α∈(0,1), implying a conservative approach. Zieliński and Zieliński (45) extend this argument using randomization, resulting in CIs with exact (1−α) coverage and minimum expected length. Alternative asymptotic CIs can be obtained by bootstrapping (e.g., refs. 46 and 47), empirical likelihood (48), sectioning (49) and other methods.

## 5. Synthetic Tasks

We illustrate the robust performance of our test and importance measure on a synthetic “known-truth” regression task implemented with neural networks. *SI Appendix*, section S2.1 provides results for a related classification task.

We consider a feature vector $X\in R^{d}$ of dimension d=19 and a response Y=μ(X)+ϵ, where the error ϵ∼N(0,1) is independent of X, and for $x=(x^{1},\ldots ,x^{d})$, the conditional mean function μ is given by

[16] $$\begin{array}{cc} \mu (x)=3 & +\;4x^{1}+x^{1}x^{2}+3{\left(x^{3}\right)}^{2}+2x^{4}x^{5}+6x^{6}+2\sin\left(x^{7}\right)\\ & +\;\exp\left(x^{8}\right)+5x^{9}+3x^{10}+4x^{11}+5x^{12}. \end{array}$$

The features $X^{1}$ and $X^{6}$ are jointly normal with mean zero, unit variances, and covariance 0.85. The features $X^{2},\ldots ,X^{5}$ and $X^{7}$ are independent N(0,1) variables, and $X^{8}$ is uniformly distributed on (−1,1). The binary feature $X^{9}=1\left\{X^{2}+W<0\right\}$

is generated from an independent W∼N(0,1). Feature $X^{10}$ follows a Poisson distribution with mean 3, while $X^{11}$ and $X^{12}$ are independent t-distributed variables with five degrees of freedom. The null variables $X^{13}$ and $X^{14}$ are independent N(0,1), $\left(X^{15},X^{16}\right)$ are jointly normal with mean zero, unit variances, and covariance 0.85, and $X^{17},X^{18}$, and $X^{19}$ are independent t-distributed variables with five degrees of freedom.

We generate a training set $I_{1}$ of $10^{6}$ independent samples $Z_{i}=\left(X_{i},Y_{i}\right)$ and a test set $I_{2}$ of $5\cdot 10^{5}$ independent samples. We train a single-layer neural network model f estimating Eq. [**16**], defined as

[17] $$\begin{array}{c} f\left(x\right)=b_{0}+\sum_{u=1}^{U}b_{u}\psi \left(a_{0,u}+a_{u}^{⊤}x\right), \end{array}$$

where U is the number of hidden units, $\psi \left(y\right)=1/\left(1+e^{-y}\right)$ is the activation function, and $b_{0},b_{u},a_{0,u}\in R$ and $a_{u}\in R^{d}$ are parameters. Training uses a standard $L_{2}$ loss criterion with $L_{2}$ regularization on the weights $b_{u}$, the Adam optimization method, a batch size of 32, and 500 epochs with early stopping. We iterate across five random initializations. A validation set (25% of $I_{1}$, excluded from training) and a Bayesian search are used to optimize the regularization weight, learning rate, and number of units U. The optimal regularization weight is $7\cdot 10^{-4}$, the optimal learning rate is $5\cdot 10^{-4}$, and the optimal number of units is U=300. The resulting model achieves an $R^{2}$ exceeding 99% on both $I_{1}$ and $I_{2}$.

Consistent with the $L_{2}$ training criterion, we use the squared loss $L\left(m,y\right)={\left(m-y\right)}^{2}$ in Eq. **6**. We adopt the masking and unmasking functions described in *Example* 2.1, yielding a conditional feature effect [**6**]. For continuous features, the masking reference value is set to the feature mean over the training set $I_{1}$ (see *SI Appendix*, section S2.2 for an alternative choice). For discrete features (ℓ=9,10), we use the adjusted mode over $I_{1}$, as specified in Section 2.2. Importantly, the validity of the test does not depend on these particular specifications. Below, we assess robustness to alternative loss functions, masking schemes, and reference values.

We run ten independent training/testing experiments to assess the performance of the test, using the default value $M_{0}=0$ for our hypotheses [**7**]. The second and third columns of Table 1 report results for significance levels α=0.05 and 0.01.^**^ Across all experiments, the fitted model and the AICO test identify as significant each feature that enters the data-generating process (DGP) through [**16**], while no feature that is null in the DGP is identified as significant. Feature correlation (e.g., ℓ=1,6), discrete feature distributions (e.g., ℓ=9,10), and interaction effects (e.g., ℓ=2,4,5) do not impair performance.

**Table 1.** Results for 10 independent training/testing experiments at two standard significance levels (α=5% and α=1%)

	AICO		CPT		HRT		LOCO		Deep knockoff		Gaussian knockoff	
*ℓ*	5%	1%	5%	1%	5%	1%	5%	1%	5%	1%	5%	1%
1	10	10	10	10	10	10	10	10	10	10	10	10
2	10	10	10	10	10	10	10	10	10	10	10	10
3	10	10	10	10	10	10	10	10	10	10	10	10
4	10	10	10	10	10	10	10	10	8	8	10	10
5	10	10	10	10	10	10	10	10	8	8	10	10
6	10	10	10	10	10	10	10	10	10	10	10	10
7	10	10	10	10	10	10	10	10	10	10	10	10
8	10	10	10	10	10	10	10	10	9	9	10	10
9	10	10	10	10	10	10	10	10	10	10	10	10
10	10	10	10	10	10	10	10	10	10	10	10	10
11	10	10	10	10	10	10	10	10	10	10	10	10
12	10	10	10	10	10	10	10	10	10	10	10	10
13	0	0	0	0	0	0	0	0	3	3	4	4
14	0	0	1	0	1	0	1	1	2	2	3	3
15	0	0	2	1	1	0	0	0	0	0	4	4
16	0	0	1	0	1	1	1	0	1	1	3	3
17	0	0	0	0	0	0	0	0	2	2	3	3
18	0	0	0	0	1	0	0	0	0	0	2	2
19	0	0	1	0	0	0	0	0	0	0	2	2

The table compares AICO, CPT, HRT, LOCO, Deep Knockoff, and Gaussian Knockoff procedures. For each feature, we report the total number of rejections across 10 trials. The test set size is $N=5\cdot 10^{5}$.

We conduct an extensive set of additional robustness experiments. Results from 2,000 independent trials, reported in *SI Appendix*, section S2.2, confirm the strong statistical performance documented in Table 1. Performance remains stable under alternative masking reference values—for example, using the mean instead of the adjusted mode for discrete features. Similarly, results are robust to the choice of loss function L, including the use of absolute rather than squared loss. Performance is, however, moderately sensitive to the masking specification: Replacing the conditional formulation [**4**] with the unconditional specification [**5**] makes features ℓ=2,4,5 less likely to be identified as significant and increases the number of discoveries among features that are null in the DGP. These features affect the response primarily through interaction terms, making their contribution harder to detect under the unconditional formulation. This behavior underscores the importance of conditioning via [**4**] when features contribute through interactions and illustrates how the masking choice influences testing in nonlinear settings.

Finally, we assess robustness with respect to the distributions of the features. The results remain stable when replacing the Gaussian distributions of features ℓ=1,…,7 with log-normal, exponential, and Beta distributions. The results are also robust to varying the correlation between the jointly Gaussian features ℓ=1,6 between 0.25 and 1. To further analyze the behavior under feature correlation, we consider a specification in which feature 1 and the DGP-null feature 13 are jointly Gaussian with correlation varying between 0.25 and 1 (while ℓ=1,6 are independent). Performance remains robust unless the correlation is close to 1, in which case the model can no longer reliably distinguish between the two features, and feature 13, although null in the DGP, is increasingly identified as significant. In the degenerate case of perfect correlation, the two features are identical and both are identified as significant.

We also evaluate the procedure in a cross-fitting framework with five (rather than one) data splits, allowing each observation to contribute to both training and testing while preserving valid inference. *SI Appendix*, section S3 provides details; *SI Appendix*, Table S9 confirms that the results remain stable under this more efficient data-splitting strategy.

We contrast our test with several recently developed model-agnostic alternatives, including the Leave-Out-Covariates Test (LOCO) of ref. 3, the Conditional Permutation Test (CPT) of ref. 1, and the Holdout Randomization Test (HRT) of ref. 2. LOCO evaluates the change in predictive performance induced by removing a feature from the model. Testing all features requires retraining the neural network d=19 times, once for each feature subset $X^{-\ell }=\left(X^{1},\ldots ,X^{\ell -1},X^{\ell +1},\ldots ,X^{d}\right)$, ℓ∈[d].^††^ The CPT and HRT procedures build on the Conditional Randomization Test of ref. 4 and evaluate the conditional independence hypothesis $Y⫫X^{\ell }|X^{-\ell }$. Both methods require sampling from the (unknown) conditional law of $X^{\ell }$ given $X^{-\ell }$. Following ref. 2, we fit d=19 mixture-density neural networks to approximate these conditional distributions.

Table 1 reports results for these alternative procedures across ten independent training/testing experiments. CPT, HRT, and LOCO identify as significant the same features as AICO in all experiments, but more frequently identify as significant features that are null in the DGP. *SI Appendix*, Table S7 reports results for 1,000 independent trials, confirming that, overall, AICO produces the fewest such discoveries among the testing procedures considered.

For additional perspective, we implement two prominent model-agnostic variable-selection procedures: the Gaussian Model-X Knockoff method of ref. 4 and the Deep Model-X Knockoff method of ref. 5. The Gaussian variant assumes a multivariate normal distribution for the features, whereas the deep variant employs a neural network to learn a generative model of the feature distribution. As shown in Table 1, the Gaussian method consistently selects features 1 through 12 in every experiment, though it also frequently selects features that are null in the DGP, whereas the Deep method selects fewer such features but shows modest difficulty identifying features 4 and 5 that enter the response function [**16**] through interaction terms.

We examine the performance of the AICO procedure as a function of the test-set size $N=|I_{2}|$. Table 2 shows that all features entering the DGP are identified as significant for sample sizes as small as N=500. For smaller N, identification rates decline moderately, while features that are null in the DGP are identified as significant only slightly more often. These results indicate reliable performance even in small-sample settings.

**Table 2.** AICO test results for 10 independent training/testing experiments, evaluated at two significance levels (α=5% and 1%) for several test sample sizes N

AICO												
	$N=10^{5}$		$N=10^{4}$		$N=10^{3}$		N=500		N=250		N=100	
*ℓ*	5%	1%	5%	1%	5%	1%	5%	1%	5%	1%	5%	1%
1	10	10	10	10	10	10	10	10	10	10	10	10
2	10	10	10	10	10	10	10	10	10	9	8	5
3	10	10	10	10	10	10	10	10	10	10	10	10
4	10	10	10	10	10	10	10	10	10	10	9	9
5	10	10	10	10	10	10	10	10	10	10	10	9
6	10	10	10	10	10	10	10	10	10	10	10	10
7	10	10	10	10	10	10	10	10	10	10	10	10
8	10	10	10	10	10	10	10	10	9	8	8	7
9	10	10	10	10	10	10	10	10	10	10	10	10
10	10	10	10	10	10	10	10	10	10	10	10	10
11	10	10	10	10	10	10	10	10	10	10	10	10
12	10	10	10	10	10	10	10	10	10	10	10	10
13	0	0	0	0	0	0	0	0	2	1	0	0
14	0	0	0	0	0	0	1	0	0	0	1	0
15	0	0	1	0	1	1	0	0	1	0	1	0
16	0	0	0	0	0	0	1	1	0	0	1	0
17	1	0	0	0	1	0	0	0	0	0	0	0
18	0	0	1	0	0	0	0	0	0	0	0	0
19	0	0	1	0	0	0	0	0	0	0	2	2

For each feature, we report the number of times the null was rejected out of 10 trials.

The experiments above reference the DGP and therefore do not directly assess the finite-sample validity result of *Proposition* 3.2. A separate numerical exercise in *SI Appendix*, section S2.4, conducted on fitted models satisfying the AICO null $M^{\ell }\leq 0$, yields rejection frequencies consistent with the exact finite-sample size property [**10**].

We now turn to measuring feature importance. Table 3 presents a feature-importance ranking based on the score [**13**] for one randomly selected training/testing experiment out of ten. It also reports the endpoints of the randomized CI [**14**] at the 99% level, along with the standard two-sided interval [**15**] and their corresponding coverage probabilities. In addition, Table 3 provides the distribution of the randomized P-values.^‡‡^

**Table 3.** AICO feature importance ranking according to the empirical median [**13**] for one of the independent training/testing experiments that was randomly chosen

AICO, Level α=1%											
				P-value distribution		Randomized one-sided CI			Nonrandomized two-sided CI		
Rank	Feature	Median	P(reject)	Lower	Upper	98.8%	1.2%	Coverage	Lower	Upper	Coverage
1	$X_{9}$	24.993	1.000	0.000	0.000	24.951	24.951	0.99	24.947	25.040	0.99007
2	$X_{6}$	15.788	1.000	0.000	0.000	15.661	15.661	0.99	15.647	15.938	0.99007
3	$X_{10}$	14.572	1.000	0.000	0.000	14.511	14.511	0.99	14.506	14.640	0.99007
4	$X_{12}$	12.898	1.000	0.000	0.000	12.789	12.789	0.99	12.775	13.022	0.99007
5	$X_{11}$	8.137	1.000	0.000	0.000	8.063	8.063	0.99	8.054	8.218	0.99007
6	$X_{1}$	6.210	1.000	0.000	0.000	6.155	6.155	0.99	6.148	6.273	0.99007
7	$X_{3}$	1.697	1.000	0.000	0.000	1.669	1.669	0.99	1.666	1.730	0.99007
8	$X_{7}$	0.953	1.000	0.000	0.000	0.941	0.941	0.99	0.940	0.964	0.99007
9	$X_{4}$	0.416	1.000	0.000	0.000	0.409	0.409	0.99	0.409	0.424	0.99007
10	$X_{5}$	0.416	1.000	0.000	0.000	0.409	0.409	0.99	0.408	0.424	0.99007
11	$X_{8}$	0.143	1.000	0.000	0.000	0.140	0.140	0.99	0.139	0.146	0.99007
12	$X_{2}$	0.084	1.000	0.000	0.000	0.082	0.082	0.99	0.082	0.086	0.99007
–	$X_{13}$	−1.154e-07	0.000	0.880	0.881	−2.442e-06	−2.437e-06	0.99	−2.708e-06	1.478e-06	0.99007
–	$X_{14}$	−2.054e-07	0.000	0.928	0.928	−2.176e-06	−2.175e-06	0.99	−2.389e-06	9.014e-07	0.99007
–	$X_{15}$	−3.060e-07	0.000	0.845	0.846	−3.339e-06	−3.338e-06	0.99	−3.639e-06	2.127e-06	0.99007
–	$X_{16}$	−5.512e-07	0.000	0.937	0.937	−2.966e-06	−2.965e-06	0.99	−3.275e-06	9.626e-07	0.99007
–	$X_{17}$	−1.340e-06	0.000	0.996	0.997	−3.773e-06	−3.773e-06	0.99	−4.022e-06	0.000	0.99150
–	$X_{18}$	0.000	0.000	0.860	0.861	−1.426e-06	−1.423e-06	0.99	−1.634e-06	1.114e-06	0.99007
–	$X_{19}$	0.000	0.000	0.815	0.816	−1.517e-06	−1.516e-06	0.99	−1.716e-06	1.333e-06	0.99007

The table also reports the endpoints of the randomized CI [**14**] for the median at a 99% level, the standard (nonrandomized) two-sided CI [**15**] along with coverage probabilities, and the distribution of the randomized P-value. Percentage heads: probabilities of the two possible lower endpoints of the randomized CI.

The P-value distributions provide a clear separation between features identified as significant and those for which the null is retained. For features 1 to 12, the distributions are concentrated near zero, far below the level α=1%. This provides strong evidence against the null hypothesis for these features, with rejection probability equal to one. In contrast, the P-value distributions for features 13,…,19 are concentrated well above α, providing clear support for retaining the null. No equivocal cases are observed, eliminating the need for randomization.

Feature 9 emerges as the most important variable, followed by features 6, 10, and 12. The remaining features are relatively less influential compared with this group. Features 4 and 5 exhibit (almost) identical medians and CIs, consistent with their symmetric roles.

As shown in Table 4, the AICO scores [**13**] and resulting rankings are stable across independent training/testing experiments, demonstrating robustness to sampling variability. Table 4 also reports the corresponding rankings for CPT, HRT, and LOCO importance measures (1, 2, 13); Deep and Gaussian Knockoff importance measures (4, 5); permutation importance (11, 12); and Deep and Kernel SHAP importance measures (9). Different measures may induce different feature rankings, which is expected since there is generally no unique ground truth for feature importance (44). Across all measures, the symmetric roles of features 4 and 5 in Eq. **16** lead to minor variation in their relative rankings across experiments.

**Table 4.** Ranking stability across ten independent training/testing experiments

ℓ	AICO	CPT	HRT	LOCO	Deep knockoff	Gaussian knockoff	Permutation importance	Deep SHAP	Kernel SHAP
1	6	7	7	7	6	6	6	5	5
2	12	11	11	12	9	9 (9–10)	11	12	12
3	7	4	4	4	5	5	5	6	6
4	9 (9–10)	8 (8–9)	8 (8–9)	9 (9–10)	12 (11–12)	12 (11–12)	8 (8–9)	9 (9–10)	9 (9–10)
5	10 (9–10)	9 (8–9)	9 (8–9)	10 (9–10)	11 (10–12)	11 (11–12)	9 (8–9)	10 (9–10)	10 (9–10)
6	2	5	5	5	1	1	2	1	1
7	8	10	10	8	8	8	10	8	8
8	11	12	12	11	10 (10–11)	10 (9–10)	12	11	11
9	1	6	6	6	7	7	7	7	7
10	3	2	3 (2–3)	2	4	4	3	3	3
11	5	3	2 (2–3)	3	3	3	4	4	4
12	4	1	1	1	2	2	1	2	2

For each measure and trial, the 12 features entering the response function are ranked by importance among themselves (1 = most important). Each cell reports the median rank across the 10 trials and, when the rank varies across trials, the corresponding range (Min–Max) in parentheses. Ties between features within a method are broken using the mean rank so that all 12 reported ranks are distinct.

We run additional experiments to evaluate the robustness of the AICO feature-importance scores to the choice of loss function L and masking specification. Replacing squared loss with absolute loss changes the magnitude of Eq. **13** but preserves feature rankings. This is expected because both loss functions quantify prediction error, albeit on different scales. Applying the training mean instead of the adjusted mode as the masking reference value for the discrete features (ℓ=9,10) moderately decreases the score [**13**] and the relative importance of these two features. This is also expected because the observed feature value tends to be closer to the mean than to the adjusted mode, reducing the magnitude of the feature effect. While using the mean as a reference value for discrete and categorical variables is generally not recommended—due to potential out-of-support values and the reference value being too close to some feature values—this experiment highlights the importance of carefully choosing these reference values.

Switching from the conditional formulation [**4**] to the unconditional specification [**5**] affects the magnitude of the scores, while the corresponding ranking remains largely stable. As discussed above in the testing context, using Eq. **5** makes interaction features ℓ=2,4,5 less likely to be identified as significant, and this behavior is also reflected in the scores. Specifically, across all trials features ℓ=4,5 have negative scores and are never identified as significant, while the score of feature ℓ=2 is highly volatile. These findings further highlight the importance of the masking specification for importance scoring.

An additional benefit of the conditional formulation [**4**] is that the corresponding score [**13**] is relatively robust to varying correlation between significant features and between significant and null features. In the degenerate case of perfect correlation, the corresponding scores collapse to identical values. This robustness contrasts with SHAP, LOCO, and permutation importance, which can be sensitive to feature correlation (13, 14).

**Fig. 2.**
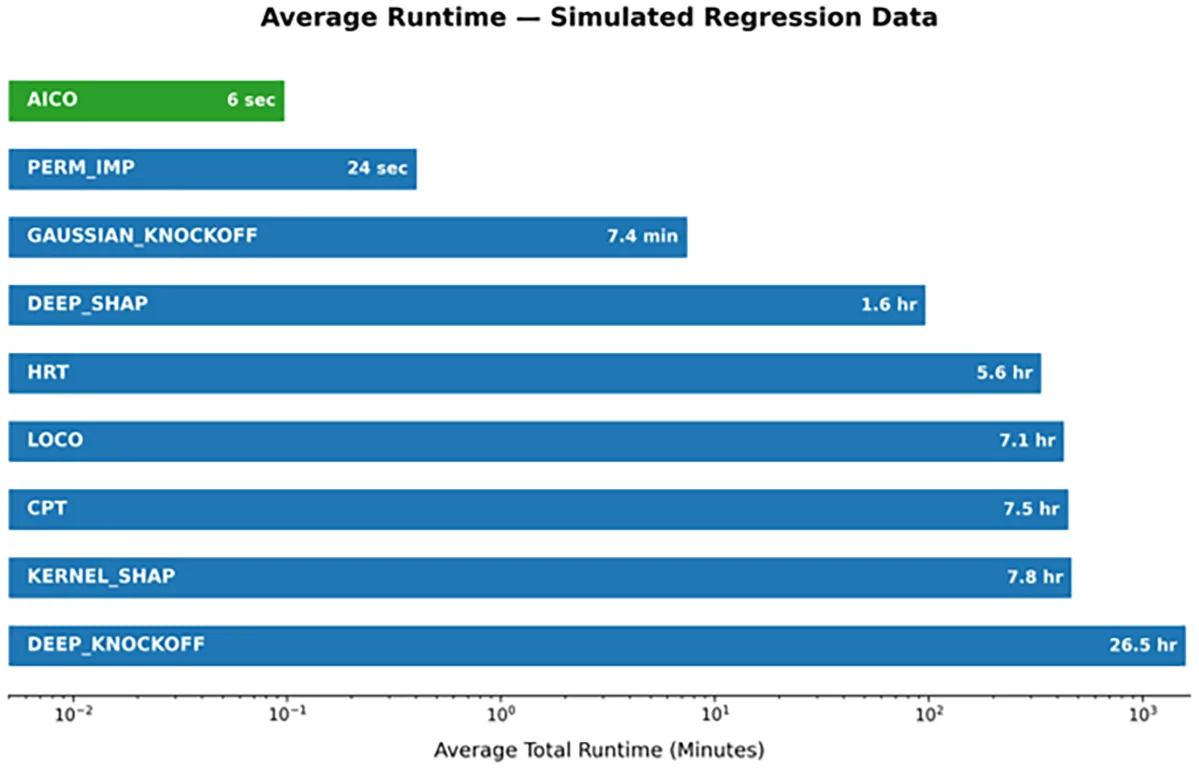
Average computation times for testing/screening all 19 features (log-scale). The compute environment consists of 8GB memory and four CPU cores (Intel Xeon CPU E5-2670, 2.60 GHz, and Silver 4110 CPU, 2.10 GHz).

Fig. 2 reports average computation times across ten experiments. In each experiment, we apply AICO, CPT, HRT, and LOCO to test all 19 features; apply the knockoff procedures to screen all 19 features; and compute SHAP and permutation importance scores for all 19 features. The reported times include any training of submodels or auxiliary models but exclude the time required to train the original neural network [**17**].

AICO achieves an exceptional degree of computational efficiency. It is more than three orders of magnitude faster than HRT, the second most efficient testing method considered. It is nearly two orders of magnitude faster than Gaussian knockoffs, the fastest screening procedure among those implemented, and four times faster than permutation importance, the most efficient algorithmic feature-importance method considered in our experiments. As shown earlier in this section, AICO’s computational efficiency does not come at the expense of statistical performance.

## 6. Empirical Applications

### 6.1. Credit Card Default.

This section complements the theoretical and numerical results with an empirical application to credit card payment data from 30,000 cardholders of a Taiwanese issuer, collected in October 2005.^§§^ The response variable is a binary indicator of payment default, and there are 23 explanatory variables (Table 5). The dataset is split into training (60%), validation (15%), and testing (25%) subsets, maintaining class proportions through stratified sampling. Categorical variables are one-hot encoded, and continuous variables are standardized to mean 0 and unit variance using training-set statistics.

**Table 5.** Raw features of the credit card default dataset

Feature	Data type	Levels	Description
SEX	Categorical	Male, Female	Sex of the cardholder
MARRIAGE	Categorical	Married, Single, Other	Marital status of the cardholder
EDUCATION	Categorical	Graduate, University, High school, Other	Education level of the cardholder
PAY_0, PAY_2–PAY_6	Categorical	Paid in full, 1…9+ months delinquent	Repayment status for the six months from Sept 2005 (PAY_0) back to Apr 2005 (PAY_6)
AGE	Continuous	–	Age of the cardholder (in years)
BILL_AMT1–BILL_AMT6	Continuous	–	Bill statement amount (NT dollars) from Sept 2005 (BILL_AMT1) back to Apr 2005 (BILL_AMT6)
LIMIT_BAL	Continuous	–	Amount of the given credit (NT dollars)
PAY_AMT1–PAY_AMT6	Continuous	–	Amount paid (NT dollars) from Sept 2005 (PAY_AMT1) back to Apr 2005 (PAY_AMT6)

We implement an XGBoost classifier (51) using a cross-entropy loss function. Model performance is evaluated by the area under the receiver operating characteristic (ROC) curve (AUC) on the validation set, which serves as the criterion for hyperparameter optimization. We use the Optuna package (52) with the Tree-Structured Parzen Estimator (53), conducting 250 trials. The search space includes the boosting algorithm, tree-specific parameters such as maximum depth and minimum child weight, and regularization terms. Class imbalance is mitigated using a scaling-weight parameter, and training is capped at 1,000 boosting rounds with early stopping after 50 rounds without improvement in validation AUC. The optimal configuration employs gradient tree boosting (54) with an approximation-based tree-building strategy, an $L_{2}$ regularization parameter of 0.2, an $L_{1}$ regularization parameter of 0.001, a subsampling ratio of 100%, a column-sampling ratio per tree of 65%, a maximum tree depth of six, a minimum child weight of two, a learning rate of 0.05, a minimum loss reduction of 0.00004, and a loss-guided tree-growth policy.

We train an ensemble of 100 XGBoost models on 100 bootstrap samples of the training data, all using the tuned hyperparameters. Ensemble predictions are obtained by averaging the outputs of all 100 models. The resulting ensemble achieves AUCs of 0.837 on the training set, 0.782 on the validation set, and 0.786 on the test set. The results are robust to the model choice; for example, an ensemble of neural networks yields nearly identical outcomes.

We adopt the same conditional feature-effect framework as in Section 5. Specifically, we use the masking and unmasking functions described in *Example* 2.1, with reference values defined by the training mean for continuous features and the adjusted training mode for discrete features.^¶¶^ We use the cross-entropy loss in Eq. **6**.

Table 6 summarizes the test results for α=1% (N=7,500 and $M_{0}=0$ in Eq. **7**). The features identified as significant, ranked by the empirical median [**13**], are PAY_0 and PAY_2 through PAY_6, MARRIAGE, SEX, and AGE. Past delinquencies are strong predictors of future payment default, with more recent payment records exerting greater influence. For actual monthly bill and payment amounts (e.g., PAY_AMT and BILL_AMT), the test does not reject the null. Marital status, sex, and age are identified as significant predictors of default even after controlling for past payment behavior. However, their influence is relatively small, especially for age: the corresponding medians, which measure effect size, are at least an order of magnitude smaller than those for the payment-history features. These findings suggest that personal circumstances exert a statistically significant, but comparatively modest, predictive influence on borrower behavior beyond patterns in prior payment.^##^

**Table 6.** AICO test summary for the credit card default dataset

AICO, Level α=1%											
				P-value distribution		Randomized one-sided CI			Nonrandomized two-sided CI		
Rank	Variable	Median	P(reject)	Lower	Upper	23.4%	76.6%	Coverage	Lower	Upper	Coverage
1	PAY_0	0.150	1.000	0.000	0.000	0.143	0.143	0.99	0.142	0.155	0.99063
2	PAY_2	0.121	1.000	0.000	0.000	0.117	0.117	0.99	0.116	0.125	0.99063
3	PAY_3	0.095	1.000	0.000	0.000	0.091	0.091	0.99	0.091	0.099	0.99063
4	PAY_4	0.059	1.000	0.000	0.000	0.055	0.055	0.99	0.054	0.064	0.99063
5	PAY_5	0.037	1.000	2.520e-322	6.423e-322	0.035	0.035	0.99	0.035	0.039	0.99063
6	PAY_6	0.030	1.000	1.020e-297	2.493e-297	0.029	0.029	0.99	0.029	0.032	0.99063
7	MARRIAGE	0.002	1.000	3.912e-11	4.561e-11	0.002	0.002	0.99	0.001	0.003	0.99063
8	SEX	0.002	1.000	1.026e-37	1.383e-37	0.001	0.002	0.99	0.001	0.002	0.99063
9	AGE	3.796e-04	1.000	1.788e-09	2.056e-09	2.200e-04	2.204e-04	0.99	2.036e-04	5.814e-04	0.99063
–	LIMIT_BAL	0.000	0.000	0.794	0.800	0.000	0.000	0.99	0.000	3.603e-04	0.99496
–	BILL_AMT1	−0.006	0.000	1.000	1.000	−0.007	−0.007	0.99	−0.008	−0.005	0.99063
–	BILL_AMT2	−0.001	0.000	1.000	1.000	−0.002	−0.002	0.99	−0.002	−9.540e-04	0.99063
–	BILL_AMT3	0.000	0.000	0.975	0.976	−1.599e-04	−1.592e-04	0.99	−1.751e-04	5.542e-05	0.99063
–	BILL_AMT4	−4.094e-04	0.000	1.000	1.000	−5.298e-04	−5.284e-04	0.99	−5.504e-04	−2.773e-04	0.99063
–	BILL_AMT5	−3.203e-04	0.000	1.000	1.000	−5.070e-04	−5.054e-04	0.99	−5.220e-04	−1.887e-04	0.99063
–	BILL_AMT6	−1.176e-04	0.000	1.000	1.000	−2.041e-04	−2.023e-04	0.99	−2.160e-04	−3.890e-05	0.99063
–	PAY_AMT1	-0.012	0.000	1.000	1.000	−0.014	−0.014	0.99	−0.014	−0.010	0.99063
–	PAY_AMT2	−0.008	0.000	1.000	1.000	−0.009	−0.009	0.99	−0.010	−0.007	0.99063
–	PAY_AMT3	−0.006	0.000	1.000	1.000	−0.006	−0.006	0.99	−0.006	−0.005	0.99063
–	PAY_AMT4	−3.764e-04	0.000	1.000	1.000	−5.811e-04	−5.779e-04	0.99	−5.998e-04	−1.935e-04	0.99063
–	PAY_AMT5	−0.002	0.000	1.000	1.000	−0.002	−0.002	0.99	−0.002	−0.002	0.99063
–	PAY_AMT6	−0.001	0.000	1.000	1.000	−0.002	−0.001	0.99	−0.002	−0.001	0.99063
–	EDUCATION	−3.891e-04	0.000	0.848	0.853	−0.001	−0.001	0.99	−0.001	4.611e-04	0.99063

Percentage heads: probabilities of the two possible lower endpoints of the randomized CI.

We can further explore the role of individual features by running the test on subsets of the test set. For instance, *SI Appendix*, Table S10 reports results for the 4,029 (out of 7,500) samples for which MARRIAGE = Single. While the test does not reject the null for EDUCATION at the 1% level, it rejects at the 5% level, as the distribution of its P-value is concentrated below 0.05. When the test is restricted to samples with MARRIAGE = Married, the null for EDUCATION is no longer rejected. These findings provide evidence that educational level contributes predictive information for unmarried borrowers, but provide no comparable evidence for married borrowers.

### 6.2. Mortgage Risk.

We next examine a more challenging classification setting, predicting the state of a mortgage loan in the next month given a history of time-varying features observed at the beginning of the month. The seven states are Current, 30 Days Delinquent, 60 Days Delinquent, 90+ Days Delinquent, Foreclosure, Paid Off, and Real Estate Owned. The analysis is based on an extensive dataset of mortgages from four ZIP codes in the greater Los Angeles area, tracked monthly from 1/1994 through 12/2022. This dataset constitutes a subset of the CoreLogic Loan-Level Market Analytics data (55). There are 52 borrower and macroeconomic features such as current loan state, credit score, loan balance, market interest rate, and local unemployment rate.

We apply the data-filtering procedure of ref. 55, resulting in roughly five million loan-month transitions from 117,523 active loans. Following ref. 55 and related work, samples with missing feature values are handled using missingness indicators and forward-filling based on the last observed value for each loan; see ref. 56 for an analysis of this approach. The training, validation, and test splits are 1/1994–6/2009, 7/2009–12/2009, and 1/2010–12/2022, respectively. We adopt the set–sequence model of ref. 6 with LongConv (57) for the sequence layer, which represents the current state of the art in mortgage transition modeling. Training and validation procedures follow those of ref. 6.

The loan-month samples form a panel dataset. *SI Appendix*, section S4 explains how such a data structure can be handled in AICO. Specifically, we follow the grouping approach in *SI Appendix*, section S4.2, treating each mortgage as a unit i with an associated group consisting of loan-month samples observed over its time horizon. Any dependence across these monthly samples is thereby contained within each group.

For testing, we use the mean multiclass cross-entropy loss, averaged over monthly time steps within each loan group. The multiclass loss accommodates all possible next-month states, while the temporal averaging enables joint testing of feature significance across all transitions in a loan’s trajectory.

We adopt the same conditional feature-effect framework as in Section 6.1. Specifically, we use the masking specification [**4**], with masking reference values $v^{\ell }$ constructed from the training set $I_{1}$ via a two-step aggregation: first from the loan-month level to the loan level, and then across loans. For each tested feature ℓ, the unit-level feature value $X_{i}^{\ell }$ is obtained by aggregating observations within unit i’s group, using the mean for continuous features and the adjusted mode for discrete and categorical features. The reference value $v^{\ell }$ in Eq. **4** is then constructed from the unit-level values $\left\{X_{i}^{\ell }:i\in I_{1}\right\}$. This two-step aggregation ensures uniform weighting across loans when constructing reference values, regardless of the number of observations available for each loan.

To avoid ambiguity, bias, and dependence on the imputation method, loan-month samples with missing values are excluded from the test set, as discussed in *SI Appendix*, section S5. Because the extent of missingness varies across features, each test is conducted on a different effective sample size. Moreover, the test is performed at the variable level, so all one-hot (dummy) features corresponding to a categorical variable are tested jointly. This yields a total of 28 tested variables.

Table 7 reports the results ($M_{0}=0$ in Eq. **7**). The most important feature by far is the current state of the mortgage. The market mortgage rate (natl_mortgage_rate) is the second most important feature, underscoring the influence of time-varying macroeconomic conditions on borrower behavior. Borrowers have a strong incentive to refinance (i.e., prepay) their mortgage when the market rate falls below their own loan rate, which is ranked ninth in importance. The results also reveal pronounced path dependence: the number of months a borrower remained current and the number of months 30 days delinquent during the past 12 months are both identified as significant and are economically important predictors of borrower behavior.

**Table 7.** AICO test summary for the mortgage risk dataset using the set-sequence model

AICO, Level α=1%													
				P-value distribution		Randomized one-sided CI				Nonrandomized two-sided CI			
Rank	Variable	Median	P(reject)	Lower	Upper	Lower-1	Lower-2	P(lwr-2)	Coverage	Lower	Upper	Coverage	Missing rate
1	current_state	0.560	1.000	0.000	0.000	0.557	0.557	0.625	0.99	0.556	0.564	0.9902	0.000
2	natl_mortgage_rate	0.006	1.000	5.372e-66	6.297e-66	0.005	0.005	0.625	0.99	0.005	0.006	0.9902	0.000
3	pool_insurance_flag	0.005	1.000	1.031e-240	1.560e-240	0.005	0.005	0.032	0.99	0.005	0.006	0.9903	0.443
4	times_current	0.002	1.000	5.920e-178	7.708e-178	0.002	0.002	0.625	0.99	0.002	0.003	0.9902	0.000
5	times_30dd_value	0.002	1.000	0.000	0.000	0.001	0.001	0.393	0.99	0.001	0.002	0.9902	0.014
6	loan_age	7.823e-04	1.000	3.830e-18	4.151e-18	5.401e-04	5.407e-04	0.625	0.99	5.230e-04	0.001	0.9902	0.000
7	collateral_type	7.683e-04	1.000	2.425e-70	2.882e-70	6.749e-04	6.750e-04	0.535	0.99	6.652e-04	8.658e-04	0.9902	0.096
8	lagged_prepay_rate	7.518e-04	1.000	7.103e-127	8.870e-127	6.730e-04	6.732e-04	0.625	0.99	6.662e-04	8.298e-04	0.9903	0.000
9	current_interest_rate	6.571e-04	1.000	8.549e-85	1.024e-84	5.789e-04	5.792e-04	0.530	0.99	5.716e-04	7.453e-04	0.9902	0.003
10	mba_days_delinquent	3.738e-04	1.000	1.890e-131	2.370e-131	3.379e-04	3.380e-04	0.625	0.99	3.341e-04	4.136e-04	0.9902	0.000
11	prepay_penalty_flag	2.733e-04	1.000	4.209e-56	4.903e-56	2.365e-04	2.366e-04	0.078	0.99	2.317e-04	3.184e-04	0.9901	0.085
12	initial_interest_rate	2.199e-04	1.000	1.219e-27	1.349e-27	1.775e-04	1.776e-04	0.625	0.99	1.751e-04	2.671e-04	0.9902	0.000
13	buydown_flag	2.192e-04	1.000	1.214e-68	1.461e-68	1.882e-04	1.883e-04	0.160	0.99	1.856e-04	2.537e-04	0.9903	0.235
14	times_foreclosure	2.032e-04	1.000	2.492e-46	2.844e-46	1.680e-04	1.681e-04	0.625	0.99	1.631e-04	2.454e-04	0.9903	0.000
15	original_balance	1.815e-04	1.000	4.651e-43	5.283e-43	1.505e-04	1.505e-04	0.625	0.99	1.479e-04	2.178e-04	0.9902	0.000
16	fico_score_at_orig	1.233e-04	1.000	4.995e-36	5.609e-36	9.650e-05	9.669e-05	0.625	0.99	9.399e-05	1.515e-04	0.9902	0.000
17	sched_monthly_pi	3.616e-05	1.000	4.464e-32	4.979e-32	2.805e-05	2.809e-05	0.067	0.99	2.688e-05	4.472e-05	0.9900	0.003
–	original_ltv	1.874e-05	0.000	0.017	0.018	−2.449e-06	−2.418e-06	0.625	0.99	−4.904e-06	4.245e-05	0.9902	0.000
–	unemployment_rate	−3.907e-04	0.000	1.000	1.000	−4.822e-04	−4.813e-04	0.625	0.99	−4.909e-04	−2.838e-04	0.9902	0.000
–	current_balance	−1.298e-06	0.000	0.520	0.524	−3.206e-05	−3.195e-05	0.625	0.99	−3.507e-05	3.639e-05	0.9902	0.000
–	sched_principal	−5.314e-05	0.000	1.000	1.000	−5.989e-05	−5.983e-05	0.980	0.99	−6.061e-05	−4.456e-05	0.9901	0.573
–	convertible_flag	−5.164e-04	0.000	1.000	1.000	−6.553e-04	−6.551e-04	0.034	0.99	−6.722e-04	−3.525e-04	0.9900	0.555
–	io_flag_value	−4.553e-05	0.000	0.973	0.973	−1.026e-04	−1.020e-04	0.868	0.99	−1.091e-04	1.431e-05	0.9902	0.324
–	neg_amortiz_flag	−8.336e-04	0.000	1.000	1.000	−0.001	−0.001	0.825	0.99	−0.001	−4.979e-04	0.9903	0.853
–	original_term	−1.041e-04	0.000	0.999	0.999	−1.843e-04	−1.843e-04	0.139	0.99	−1.924e-04	−1.752e-05	0.9900	0.0001
–	times_60dd	−3.928e-04	0.000	1.000	1.000	−5.032e-04	−5.024e-04	0.625	0.99	−5.118e-04	-2.708e-04	0.9902	0.000
–	times_90dd	−4.781e-04	0.000	1.000	1.000	−5.630e-04	−5.630e-04	0.625	0.99	−5.723e-04	−3.823e-04	0.9902	0.000
–	lagged_forecl_rate	−1.049e-04	0.000	0.998	0.998	−1.769e-04	−1.765e-04	0.625	0.99	−1.846e-04	−9.468e-06	0.9902	0.000

Training data are grouped and treated at the loan-month level. The *missing rate* denotes the ratio of feature effects dropped from the test set due to missingness to the total number of loan-level feature effects after grouping (46,890). *P(lwr-2)* represents the probability associated with the larger lower bound (*lower-2*) of the one-sided randomized CI.

## 7. Materials and Methods

This article includes *SI Appendix*; there are several sections. *SI Appendix*, section S1 provides the proofs of Propositions 3.2 and 4.1. *SI Appendix*, section S2 provides additional numerical results. *SI Appendix*, section S3 discusses a cross-fitting implementation of AICO. *SI Appendix*, section S4 explains the treatment of panel data structures. *SI Appendix*, section S5 discusses the treatment of missing feature data. *SI Appendix*, section S6 provides additional empirical results.

## Supplementary Material

Appendix 01 (PDF)

## Data Availability

Code repository data have been deposited in Github (https://github.com/ji-chartsiri/AICO) (58). Data cannot be shared (Data used in one of the empirical examples was licensed from CoreLogic Inc. The license terms do not allow us to share the data. However, interested parties can obtain a license from CoreLogic to get access to the data). Previously published data were used for this work (https://archive.ics.uci.edu/dataset/350/default+of+credit+card+clients) (59).

## Footnotes

^*^An open-source Python package is available at https://github.com/ji-chartsiri/AICO.

^†^Methods based on removing features typically require retraining the model for each feature under consideration, which is prohibitive for feature-rich models. The approach of ref. 33 requires retraining the model once using a custom training algorithm. Model-X knockoff procedures require constructing an auxiliary model of the joint feature distribution, a task that can be as challenging as fitting the predictive model itself; see ref. 35 for fundamental limitations.

^‡^Applications of sample splitting to testing problems have a long history; Cox (36) is an early reference. See refs. 29 and 34 for recent developments.

^§^In this section, all expectations are taken with respect to the (unknown) distribution of Z=(X,Y) from which the data are drawn.

^¶^Note that Eq. **2** estimates Eq. **3** at the sample level if $f(m^{\ell }\left(X\right))$, the predicted mean response after masking ℓ, approximates $E(Y|X^{-\ell })$, the true mean response after removing ℓ.

^#^Even when a reference value is marginally in the feature support, masking can still produce covariate combinations that force the model to extrapolate into regions with limited data support. The risk of such off-manifold evaluations can be mitigated through localized or conditional masking schemes that construct reference values using observations with similar feature profiles. For a concrete example, see *SI Appendix*, section S2.2.

^‖^With these specifications, L measures predictive accuracy. Other choices of L can quantify model performance in downstream screening or decision-making applications of f (e.g., revenue or cost), such as the financial loss resulting from a poor decision due to misclassification.

^**^Limited numerical precision can lead to ties, i.e., samples $\Delta^{\ell }\left(Z_{i}\right)$ whose true values are distinct being rounded to the same value. If the value of a tie is strictly positive, we count each sample in the test statistic $n_{+}^{\ell }$. Another numerical artifact arises when samples $\Delta^{\ell }\left(Z_{i}\right)$ with true, nonzero values are rounded to zero. We treat these as nonpositive, excluding them from $n_{+}^{\ell }$—a conservative choice. Alternative, potentially less conservative treatments include dropping ties (adjusting N) or randomly breaking them. See also ref. 50 for another approach.

^††^In our numerical experiments, each of the d=19 submodels has the same architecture as the full model f and is trained using the same procedure.

^‡‡^Ties among samples $\Delta^{\ell }\left(Z_{i}\right)$ due to limited numerical precision are retained in $\left\{\Delta^{\ell }\left(Z_{i}\right):i\in I_{2}\right\}$ when computing CIs and coverage probabilities. This treatment is consistent with the handling of ties in the testing procedure (footnote ^**^).

^§§^The dataset is available at https://archive.ics.uci.edu/dataset/350/default+of+credit+card+clients.

^¶¶^We treat each categorical variable as a single discrete feature rather than analyzing its associated one-hot encodings separately. If higher resolution is desired, the test can alternatively be run for each individual level of a categorical variable.

^##^In the United States, marital status, sex, and age are protected characteristics under fair lending laws and therefore are not used—indeed, usually not even collected—for estimating default probabilities or credit scores in credit underwriting.
