# Supplementary material for: AICO: Feature significance tests for supervised learning
Source: Proc Natl Acad Sci U S A. 2026 Sep 23;123(39):e2530045123. doi: 10.1073/pnas.2530045123 (PMC13624631; doi:10.1073/pnas.2530045123)
Supplement: Supplementary file 1 [file pnas.2530045123.sapp.pdf]

PNAS

Supporting Information for

AICO: Feature Significance Tests for Supervised Learning

Kay Giesecke, Enguerrand Horel, and Chartsiri Jirachotkulthorn

Kay Giesecke
E-mail: Kay.Giesecke@hpi.de

This PDF file includes:

Supporting text
Fig. S1
Tables S1 to S10
SI References

Supporting Information Text

S1. Proofs of technical results

Proof of Proposition 2.2. Due to Assumption 2.1, $n_+^\ell(w) \sim B_{N,\theta_w^\ell}$ under the sampling distribution $\mathbb{P}_{M^\ell}$, where $\theta_w^\ell = 1 - F^\ell(w)$. The Binomial distribution B_{N,θ_w^ℓ} has a monotone likelihood ratio, so the Neyman-Pearson lemma (1, Theorem 3.4.1) guarantees the existence of a uniformly most powerful (UMP) test for the hypotheses

$$\theta_w^\ell \leq \theta_0 \quad \text{vs.} \quad \theta_w^\ell > \theta_0$$

for some $\theta_0 \in [0,1]$. Due to the continuity of F^ℓ, we have $F^\ell(M^\ell) = 1/2$ so for $\theta_0 = 1/2$ and $w = M_0$ these hypotheses are equivalent to (7). The UMP test has test function $\phi(n) = 1\{n > T\} + \gamma 1\{n = T\}$ for some $T \in \mathbb{N}$ and $\gamma \in [0,1]$ determined by the requirement that the test's level of significance at the boundary $M^\ell = M_0$ be equal to α. Specifically, for any $M_0 \in \mathbb{R}$,

$$\begin{aligned}\alpha &= \mathbb{E}_{M_0}(\phi(n_+^\ell(M_0))) \\ &= \mathbb{P}_{M_0}(n_+^\ell(M_0) > T) + \mathbb{E}_{M_0}(\gamma | n_+^\ell(M_0) = T)\mathbb{P}_{M_0}(n_+^\ell(M_0) = T),\end{aligned}$$

where $\mathbb{E}_{M_0}$ represents expectation under $\mathbb{P}_{M_0}$, the sampling distribution $\mathbb{P}_{M^\ell}$ of Assumption 2.1 for $M^\ell = M_0$. Clearly $n_+^\ell(M_0) \sim B_{N,1/2}$ under $\mathbb{P}_{M_0}$. Moreover, it follows that $T = T_{N,\alpha} = q_{1-\alpha}(B_{N,1/2})$ and

$$\gamma = \gamma_N(T,\alpha) = \frac{\alpha - \mathbb{P}_{M_0}(n_+^\ell(M_0) > T)}{\mathbb{P}_{M_0}(n_+^\ell(M_0) = T)},$$

where $\mathbb{P}_{M_0}(n_+^\ell(M_0) \leq n) = \Pi_{N,1/2}(n)$ and $\mathbb{P}_{M_0}(n_+^\ell(M_0) = n) = \pi_{N,1/2}(n)$. This gives (9). □

Proof of Proposition 3.1. Let $U \sim U(0,1)$ and $I^\ell(w) = 1 - \phi_N(n_+^\ell(w),\alpha)$, where we recall $n_+^\ell(w) = \#\{i \in I_2 : \Delta^\ell(Z_i) > w\}$ for $w \in \mathbb{R}$, as in Proposition 2.2. From (2), a randomized CI for M^ℓ is given by the U-cut of $I^\ell(w)$:

$$C_N^\ell(\alpha;U) = \{w \in \mathbb{R} \mid I^\ell(w) \geq U\}.$$

Then, by iterated expectations, the independence of U, and Eq. (10) we get

$$\begin{aligned}\mathbb{P}_{M^\ell}(M^\ell \in C_N^\ell(\alpha;U)) &= \mathbb{P}_{M^\ell}(U \leq I^\ell(M^\ell)) \\ &= \mathbb{E}_{M^\ell}(1 - \phi_N(n_+^\ell(M^\ell),\alpha)) \\ &= 1 - \alpha.\end{aligned}$$

Since $n_+^\ell(M^\ell) = N - i$ for $M^\ell \in [\Delta_{(i)}^\ell, \Delta_{(i+1)}^\ell)$, we have

$$C_N^\ell(\alpha;U) = \bigcup \{[\Delta_{(i)}^\ell, \Delta_{(i+1)}^\ell) \mid i = 0,\ldots,N;\ 1 - \phi_N(N-i,\alpha) \geq U\}.$$

Moreover, note that

$$\begin{aligned}1 - \phi_N(N-i,\alpha) &= 1\{N - i \leq T_{N,\alpha}\} - \gamma_N(T_{N,\alpha},\alpha)1\{N - i = T_{N,\alpha}\} \\ &= 1\{N - T_{N,\alpha} < i\} + (1 - \gamma_N(T_{N,\alpha},\alpha))1\{N - T_{N,\alpha} = i\}.\end{aligned}$$

Thus $C_N^\ell(\alpha;U)$ is equal to the union of $[\Delta_{(N-T_{N,\alpha})}^\ell, \Delta_{(N-T_{N,\alpha}+1)}^\ell)$ and $[\Delta_{(N-T_{N,\alpha}+1)}^\ell, \infty)$ if $1 - \gamma_N(T_{N,\alpha},\alpha) \geq U$ and $[\Delta_{(N-T_{N,\alpha}+1)}^\ell, \infty)$ otherwise. □

S2. Additional experimental results

Appendix S2.1 provides numerical results for a classification setting, complementing the regression results in Section 4. Appendix S2.2 provides additional robustness results. Appendix S2.3 compares the testing performance of AICO with alternative procedures over a large number of trials. Appendix S2.4 examines the finite-sample validity of the AICO test.

S2.1. Classification. This section illustrates the robust performance of our test and feature-importance measure on a synthetic "known-truth" classification task. Consider a binary response $Y \in \{0,1\}$ governed by the logistic model

$$\mathbb{E}(Y|X) = \mathbb{P}(Y = 1|X) = g(\mu(X)), \qquad [1]$$

where $g(w) = 1/(1 + e^w)$, the function μ is given by (16), and the features $X = (X^1,\ldots,X^d)$ are defined as in Section 4. We generate a training set I_1 of 10^6 independent samples Z_i and a test set I_2 of $5 \cdot 10^5$ independent samples. The data are imbalanced, as is common in practice: approximately 10% of the samples satisfy $Y_i = 1$.

We train a single-layer neural network f estimating Eq. (1), specified as

$$f(x) = g\left(b_0 + \sum_{u=1}^{U} b_u \psi(a_{0,u} + a_u^\top x)\right), \qquad [2]$$

where $\psi(y) = 1/(1+e^{-y})$, U is the number of hidden units, and $b_0, b_u, a_{0,u} \in \mathbb{R}$ and $a_u \in \mathbb{R}^d$ are parameters. Training uses a standard cross-entropy loss with L_2 regularization (weight 10^{-3}, approximately optimal in the regression setting), the Adam optimizer, a batch size of 32, and 500 epochs with early stopping. A validation set (25% of I_1) and a Bayesian search procedure are used to tune the learning rate and number of units U. The optimal learning rate is $5 \cdot 10^{-4}$ and the optimal $U = 300$. The resulting model achieves an Area Under the ROC Curve exceeding 99% on both the training and test data.

The testing procedure employs the cross-entropy loss $L(m, y) = -(y \log m + (1-y)\log(1-m))$ in (6), consistent with the training objective. We adopt the same conditional feature-effect framework as in Section 4. Specifically, we use the masking and unmasking functions described in Example 1.1, with reference values defined by the training mean for continuous features and the adjusted training mode for discrete features.

Table S1 reports the results for significance levels $\alpha = 0.01$ and 0.05 across ten independent experiments, again using the default value $M_0 = 0$ in (7). Across all experiments, features entering the data-generating process (DGP) are identified as significant, while features that are null in the DGP are identified as significant only occasionally. Feature correlation (e.g., $\ell = 1, 6$), discrete feature distributions (e.g., $\ell = 9, 10$), and interaction effects (e.g., $\ell = 2, 4, 5$) do not impair performance.

ℓ	AICO		CPT		HRT		LOCO		Deep Knockoff		Gaussian Knockoff	
	5%	1%	5%	1%	5%	1%	5%	1%	5%	1%	5%	1%
**1**	10	10	10	10	10	10	10	10	10	10	10	10
**2**	10	10	10	10	10	10	10	10	10	10	10	10
**3**	10	10	10	10	10	10	10	10	10	10	10	10
**4**	10	10	10	10	10	10	10	10	9	9	10	10
**5**	10	10	10	10	10	10	10	10	10	10	9	9
**6**	10	10	10	10	10	10	10	10	10	10	10	10
**7**	10	10	10	10	10	10	10	10	10	10	10	10
**8**	10	10	10	10	10	10	10	10	9	9	9	9
**9**	10	10	10	10	10	10	10	10	10	10	10	10
**10**	10	10	10	10	10	10	10	10	10	10	6	6
**11**	10	10	10	10	10	10	10	10	10	10	10	10
**12**	10	10	10	10	10	10	10	10	10	10	10	10
**13**	1	1	0	0	0	0	4	4	2	2	1	1
**14**	0	0	2	0	0	0	5	5	1	1	1	1
**15**	0	0	1	0	2	0	6	6	0	0	3	3
**16**	0	0	1	1	2	1	5	5	1	1	2	2
**17**	1	1	0	0	0	0	5	5	0	0	1	1
**18**	1	1	1	0	0	0	5	5	0	0	2	2
**19**	1	1	0	0	1	1	3	3	0	0	1	1

Table S1. Classification model test results for 10 independent training/testing experiments at two standard significance levels ($\alpha = 5\%$ and $\alpha = 1\%$). The table compares AICO, CPT, HRT, LOCO, Deep Knockoff, and Gaussian Knockoff procedures, showing the total number of rejections across 10 trials. The test set size is $N = 5 \cdot 10^5$.

To assess robustness, we perform 2,000 additional independent trials, reported in S2.2. These experiments corroborate the performance shown in Table S1. Features entering the DGP continue to be identified as significant with high frequency, while features that are null in the DGP are generally identified as significant more frequently than in the regression setting.

The classification task appears to be more challenging than the regression task in this setting. Whereas the regression model estimates a smooth conditional mean function, the classification model must learn a nonlinear decision boundary separating classes. Consequently, the fitted classifier is more sensitive to finite-sample variation and more likely to assign predictive value to features that are null in the DGP. This behavior helps explain why features that are null in the DGP are identified as significant somewhat more frequently than in the regression setting.

The procedure remains stable under alternative choices of the loss function L, including the use of squared loss in place of cross-entropy loss. Greater sensitivity arises with respect to the masking specification. Specifically, using the mean rather than the adjusted mode for masking discrete features makes the Poisson-distributed feature $\ell = 10$ less likely to be identified as significant. This occurs because the mask often coincides with, or lies close to, the feature value being masked, rendering the masking operation less effective. The effect is particularly pronounced in the classification setting, where the model output is compressed to $[0, 1]$.

Replacing the conditional formulation (4) with the unconditional specification (5) makes features $\ell = 2, 4, 5$ less likely to be identified as significant and increases the frequency with which features that are null in the DGP are identified as significant. In particular, features $\ell = 4, 5$ enter the response function through an interaction term, making their contribution harder to isolate under the unconditional formulation. This behavior, also observed in the regression case (Section 4), highlights the importance of conditioning when features influence the response primarily through interactions.

Table S1 also reports results for CPT, HRT, LOCO, and the knockoff procedures. AICO, CPT, HRT, and LOCO consistently identify as significant all features entering the DGP, while the knockoff approaches show modest difficulty identifying several

such features. LOCO, however, identifies as significant features that are null in the DGP substantially more frequently than the other methods. A plausible explanation is that finite-sample estimation noise prevents the fitted model from being exactly invariant to DGP-null features. Since LOCO compares predictions from two independently fitted models (the full model and a reduced model retrained without the candidate feature), small discrepancies between the fits may not fully cancel across test samples and can propagate into the test statistic. The effect appears more pronounced in the classification setting, where the prediction problem is more difficult and the resulting finite-sample discrepancies between the fitted models may be larger. This mechanism may contribute to the higher frequency with which LOCO identifies as significant features that are null in the DGP.

	**AICO**											
ℓ	$N = 10^5$		$N = 10^4$		$N = 10^3$		$N = 500$		$N = 250$		$N = 100$	
	5%	1%	5%	1%	5%	1%	5%	1%	5%	1%	5%	1%
**1**	10	10	10	10	10	10	10	10	9	7	3	0
**2**	10	10	10	10	6	5	3	2	6	5	3	1
**3**	10	10	10	10	10	10	10	10	10	10	8	8
**4**	10	10	10	10	10	10	10	9	6	2	4	0
**5**	10	10	10	10	10	10	8	8	8	6	6	1
**6**	10	10	10	10	10	10	10	10	8	6	2	1
**7**	10	10	8	7	3	3	2	0	1	0	0	0
**8**	10	10	5	2	0	0	0	0	0	0	0	0
**9**	10	10	10	10	6	3	3	1	5	3	3	1
**10**	10	10	10	10	10	10	10	10	9	9	8	5
**11**	10	10	10	10	6	2	9	5	0	0	3	1
**12**	10	10	10	10	10	9	6	5	5	2	2	0
**13**	0	0	1	0	1	0	3	0	0	0	0	0
**14**	0	0	0	0	0	0	1	0	1	0	2	1
**15**	0	0	0	0	0	0	0	0	1	1	1	0
**16**	0	0	0	0	0	0	0	0	0	0	2	0
**17**	1	1	1	0	1	0	2	0	0	0	0	0
**18**	1	1	1	0	1	0	2	0	0	0	0	0
**19**	0	0	1	0	1	0	0	0	0	0	0	0

Table S2. Classification model test results for 10 independent training/testing experiments, at two significance levels ($\alpha = 5\%$ and $\alpha = 1\%$), across various test sample sizes N. We report the total number of rejections over 10 trials.

Table S2 reports test results for varying test-set sample sizes N. As N decreases, identification rates for features entering the DGP decline more rapidly than in the regression case, particularly for features $\ell = 7$ and $\ell = 8$. The frequency with which features that are null in the DGP are identified as significant appears largely insensitive to N.

As in the regression setting, the experiments above reference the DGP rather than the AICO null. A separate numerical demonstration of the finite-sample size property (10) is provided in Appendix S2.4.

Table S3 presents a feature-importance ranking based on the empirical median (13) for one randomly chosen training/testing experiment out of ten. It also reports the endpoints of the randomized confidence interval (14) for the population median M at the 99% level, together with the standard (non-randomized) two-sided interval (15) and their coverage probabilities. Table S3 further shows the distribution of the randomized p-values.

As shown in Table S4, the AICO scores (13) and importance rankings are relatively stable across independent training/testing experiments. Table S4 also reports the corresponding rankings for CPT, HRT, and LOCO importance measures; Deep and Gaussian Knockoff importance measures; permutation importance; and Deep and Kernel SHAP importance measures. Compared with the regression setting, the rankings are somewhat more variable across experiments for all measures, reflecting the greater difficulty of the classification task. Across all measures, the symmetric roles of features 4 and 5 again lead to minor variation in their relative rankings across experiments.

The choice of loss function L and masking specification can affect the scores (13) and the resulting feature importance rankings. The behavior is qualitatively similar to that in the regression case. In particular, switching from cross-entropy loss to squared loss reduces the magnitude of (13) but preserves the rankings. While not recommended, choosing the mean rather than the adjusted mode as the masking reference value for the discrete features $\ell = 9, 10$ also reduces the corresponding median values.

Fig. S1 reports average computation times across ten experiments. As in the regression case, AICO achieves exceptional computational efficiency. Among the testing procedures considered here, it is more than three orders of magnitude faster than LOCO, the next most efficient method. AICO is more than an order of magnitude faster than Gaussian knockoffs, the fastest screening approach among those implemented, and six times faster than permutation importance, the most efficient algorithmic feature-importance method considered in our experiments.

We also evaluate the procedure in a cross-fitting framework with five data splits. Table S9 below confirms that features entering the DGP continue to be identified as significant with high frequency under this more efficient data-splitting strategy, although features that are null in the DGP are identified as significant slightly more frequently.

AICO, Level $\alpha = 1\%$											
				p-value Distribution		Randomized One-Sided CI			Non-Randomized Two-Sided CI		
Rank	Feature	Median	P(reject)	Lower	Upper	98.8%	1.2%	Coverage	Lower	Upper	Coverage
1	X_3	3.577e-07	1.000	0.000	0.000	3.340e-07	3.340e-07	0.99	3.320e-07	3.829e-07	0.99007
2	X_{10}	2.324e-08	1.000	0.000	0.000	2.077e-08	2.077e-08	0.99	2.053e-08	2.619e-08	0.99007
3	X_6	6.722e-10	1.000	0.000	0.000	6.014e-10	6.016e-10	0.99	5.939e-10	7.656e-10	0.99007
4	X_1	1.630e-10	1.000	0.000	0.000	1.442e-10	1.443e-10	0.99	1.426e-10	1.862e-10	0.99007
5	X_{12}	6.989e-11	1.000	0.000	0.000	5.826e-11	5.828e-11	0.99	5.702e-11	8.597e-11	0.99007
6	X_5	4.339e-11	1.000	0.000	0.000	3.697e-11	3.697e-11	0.99	3.643e-11	5.135e-11	0.99007
7	X_4	3.169e-11	1.000	0.000	0.000	2.707e-11	2.707e-11	0.99	2.668e-11	3.733e-11	0.99007
8	X_{11}	1.001e-11	1.000	0.000	0.000	8.027e-12	8.030e-12	0.99	7.821e-12	1.275e-11	0.99007
9	X_9	6.787e-12	1.000	0.000	0.000	5.025e-12	5.025e-12	0.99	4.863e-12	9.439e-12	0.99007
10	X_2	2.575e-14	1.000	0.000	0.000	1.823e-14	1.825e-14	0.99	1.755e-14	3.704e-14	0.99007
11	X_7	1.100e-15	1.000	3.262e-71	3.431e-71	5.257e-16	5.261e-16	0.99	4.808e-16	2.326e-15	0.99007
12	X_8	1.344e-17	1.000	1.706e-14	1.743e-14	2.386e-18	2.394e-18	0.99	1.972e-18	4.921e-17	0.99007
-	X_{13}	0.000	0.000	1.000	1.000	0.000	0.000	0.99	0.000	0.000	1.00000
-	X_{14}	0.000	0.000	1.000	1.000	-1.396e-21	-1.390e-21	0.99	-2.680e-21	0.000	0.99485
-	X_{15}	-3.193e-21	0.000	1.000	1.000	-5.676e-20	-5.672e-20	0.99	-7.258e-20	0.000	0.99503
-	X_{16}	0.000	0.000	1.000	1.000	-3.066e-22	-3.052e-22	0.99	-5.372e-22	0.000	0.99501
-	X_{17}	0.000	0.000	1.000	1.000	0.000	0.000	0.99	0.000	0.000	0.99992
-	X_{18}	0.000	0.000	0.940	0.940	0.000	0.000	0.99	-1.849e-32	1.780e-22	0.99007
-	X_{19}	0.000	0.000	1.000	1.000	0.000	0.000	0.99	0.000	0.000	0.99999

Table S3. Classification model feature importance ranking according to the empirical median (13) for one of the independent training/testing experiments that was randomly chosen. The table also reports the endpoints of the randomized CI (14) for the median at a 99% level, the standard (non-randomized) two-sided CI (15) along with coverage probabilities, and the distribution of the randomized p-value. Percentage heads: probabilities of the two possible lower endpoints of the randomized CI.

S2.2. AICO test results for large number of trials. Table S5 reports AICO test results for 2,000 independent experiments for the regression task (Section 4) and the classification task (Appendix S2.1). These results complement the results reported in columns two and three of Tables 1 and S1 for ten independent experiments. The Bonferroni-adjusted counts in panel (b) demonstrate the effectiveness of standard multiplicity corrections in our setting.

Several DGP-null features exhibit relatively elevated rejection counts. These include the correlated pair $(15, 16)$ in the regression model and several features in the classification model. Moreover, in the classification model, several features entering the DGP have somewhat reduced rejection counts. An additional experiment suggests that variability induced by random initialization during training contributes to both phenomena. It also indicates that the elevated counts for the correlated pair $(15, 16)$ are amplified by off-manifold extrapolation induced by marginal masking reference values.

The variability due to random initialization can be reduced through model averaging (ensembling), which generalizes the best-model strategy used previously (selecting the model, or random initialization, performing best on the validation set). The extrapolation issue arises for the correlated pair $(15, 16)$ under marginal masking. Marginal masking sets X^{15} to its mean while X^{16} retains its observed value, sending the input into a region the training data cover only sparsely (since in training $X^{15} \approx 0.85 \cdot X^{16}$). The model must then extrapolate, which can induce positive feature effects and increase the frequency with which the feature is identified as significant. This is fundamentally a fitting and sampling issue: a given realization of D_1 may cover that region especially poorly, causing the fitted model to extrapolate unreliably there. To address this issue, we replace the marginal-mean reference value with a conditional mean given $X^{-\ell}$, estimated by ordinary least squares of X^{ℓ} on the remaining features.* This choice tends to keep the masked input in a more densely covered region of the feature space and substantially reduces the rejection counts on X^{15} and X^{16} while preserving the ability to identify the DGP-relevant features.

Table S6 reports the 2,000-trial experiment with fresh training and test data drawn in each trial, using a best-three-of-five ensemble (five networks trained per trial from independent initializations, the three with the lowest validation loss averaged) and the conditional-mean reference value, in which each continuous feature is masked by its conditional mean given the remaining features, estimated from the training data by ordinary least squares. The results demonstrate that combining the ensembling strategy with the conditional-mean reference value substantially reduces the rejection counts for DGP-null features and increases the counts for features entering the DGP. As shown in panel (b) of Table S6, a standard Bonferroni adjustment further reduces rejection counts for DGP-null features while only minimally affecting rejection counts for features entering the DGP.

The rejection counts for DGP-null features in Table S6 are moderately higher in the classification setting than in the regression setting. This suggests that DGP-null features in the classification model more frequently induce median feature effects close to the AICO boundary value $M^{\ell} = 0$. Near this boundary, the rejection probability approaches the nominal significance level, so relatively small shifts in the feature-effect distribution due to fitting and sampling variability can increase the frequency with which DGP-null features are identified as significant. A plausible explanation is that the classification

*An alternative to the conditional mean is a nearest-neighbor estimator.

ℓ	AICO	CPT	HRT	LOCO	Deep Knockoff	Gaussian Knockoff	Permutation Importance	Deep SHAP	Kernel SHAP
**1**	4	7	7	5 (5–7)	4 (3–4)	3	5	5	5
**2**	10	11	11	11 (9–12)	7	6 (6–7)	11	9 (9–10)	9 (9–11)
**3**	1	5	5	6 (5–6)	6	5	6	7	7
**4**	7 (6–9)	9 (8–10)	9 (8–10)	10 (7–11)	10 (9–12)	10 (9–12)	9 (8–10)	12 (11–12)	12 (10–12)
**5**	6 (5–8)	8 (8–9)	8 (8–9)	9 (8–11)	11 (9–12)	12 (10–12)	8 (8–9)	11 (11–12)	11 (11–12)
**6**	3	4	4	4	1 (1–2)	1	2	1 (1–2)	1 (1–2)
**7**	11	10 (8–10)	10 (8–10)	8 (7–12)	8 (8–9)	8 (7–9)	10 (8–10)	8	8
**8**	12	12	12	12 (10–12)	12 (10–12)	11 (9–12)	12	10 (9–10)	10 (9–10)
**9**	9 (7–9)	6	6	7 (6–11)	9 (8–12)	7 (6–9)	7	6	6
**10**	2	3	3	3	5	9 (6–12)	4	3 (3–4)	3
**11**	8 (6–9)	2	2	2	3 (3–4)	4	3	4 (3–4)	4
**12**	5 (5–7)	1	1	1	2 (1–2)	2	1	2 (1–2)	2 (1–2)

Table S4. Ranking stability across ten independent training/testing experiments. For each measure and trial, the 12 significant features are ranked by importance among themselves (1 = most important). Each cell reports the median rank across the 10 trials and, when the rank varies across trials, the corresponding range (Min–Max) in parentheses. Ties between features within a method are broken using the mean rank so that all 12 reported ranks are distinct.

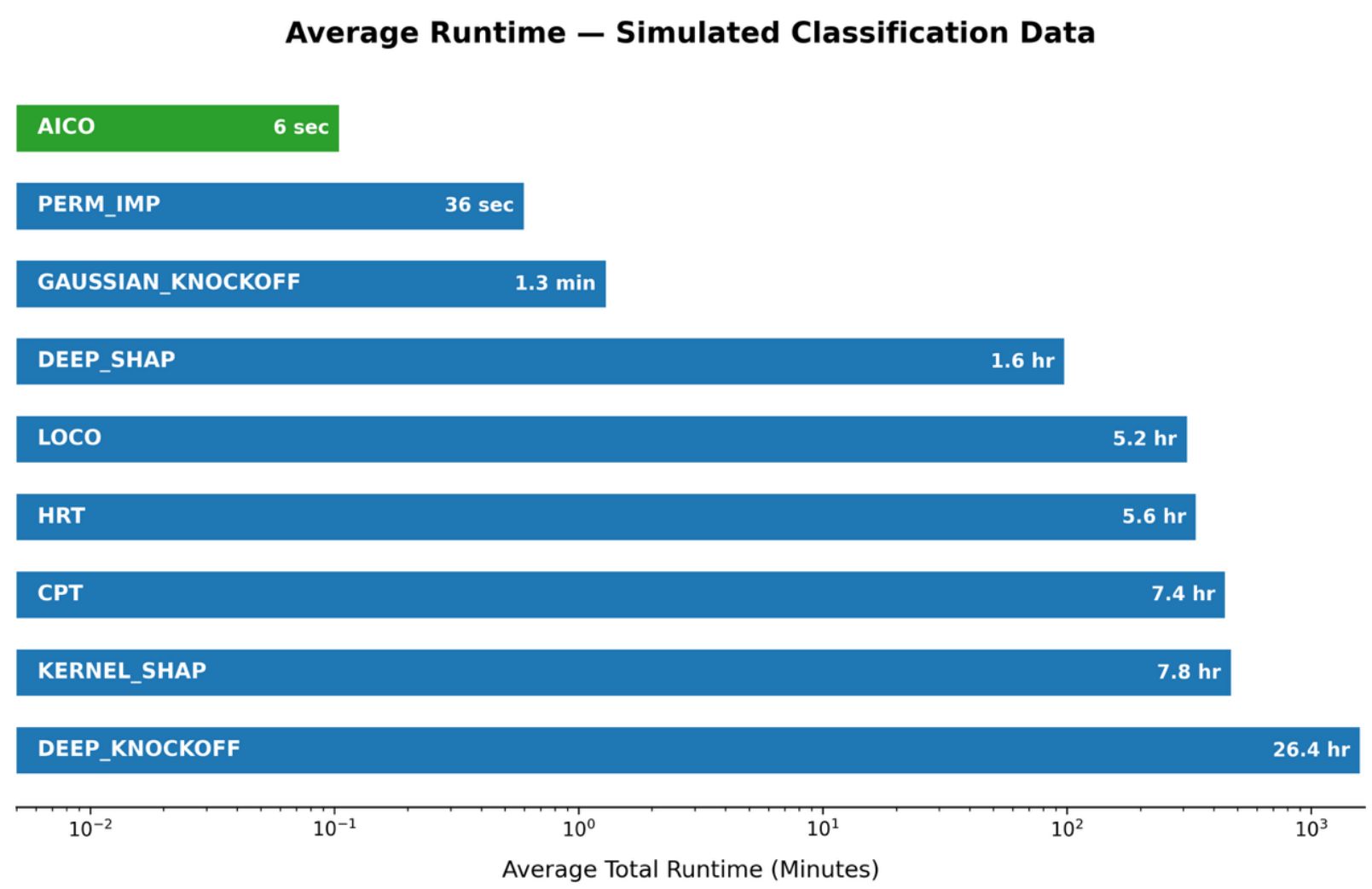

Fig. S1. Average computation times for testing/screening all 19 features (log-scale). The compute environment consists of 8GB memory and four CPU cores (Intel Xeon CPU E5-2670, 2.60GHz, and Silver 4110 CPU, 2.10GHz).

problem is intrinsically more difficult than the regression problem, making the fitted model more sensitive to finite-sample variation.

S2.3. Comparative testing performance for large numbers of trials. Tables 1 and S1 compare the performance of AICO, CPT, HRT, and LOCO across ten independent training/testing experiments. Table S7 below reports results for 1,000 independent experiments for the regression model (16). The setting mirrors that of Section 4, with one exception: to accommodate the substantially longer computation times of CPT, HRT, and LOCO (see Fig. 2), the training set size was reduced from 10^6 to 10^4 and the test set size was reduced from $5 \cdot 10^5$ to $N = 5 \cdot 10^3$. The results confirm the strong performance of the AICO procedure in a setting with relatively small training and testing samples.

All procedures identify as significant the features entering the DGP in every experiment, but there are substantial differences in how frequently features that are null in the DGP are identified as significant. Among the methods considered, AICO exhibits the lowest such frequencies overall, by a considerable margin. The corresponding frequencies for CPT and HRT are moderately higher, while those for LOCO are substantially higher, consistent with the behavior observed in the smaller-scale experiments (see Table S1).

S2.4. Finite-sample validity of AICO test. Equation (10) establishes the exact finite-sample validity of the AICO test: conditional on the training data D_1, a feature with median effect $M^\ell \le M_0$ is rejected with probability at most α, with equality attained only at the boundary $M^\ell = M_0$. We demonstrate this property numerically for the regression task (Section 4) and the

ℓ	AICO Regression 5%	1%	AICO Classification 5%	1%
**1**	2000	2000	2000	2000
**2**	2000	2000	2000	2000
**3**	2000	2000	1872	1872
**4**	2000	2000	1851	1851
**5**	2000	2000	1847	1846
**6**	2000	2000	2000	2000
**7**	2000	2000	2000	2000
**8**	2000	2000	2000	2000
**9**	2000	2000	2000	2000
**10**	2000	2000	2000	2000
**11**	2000	2000	2000	2000
**12**	2000	2000	2000	2000
**13**	0	0	43	21
**14**	1	0	56	32
**15**	117	31	59	33
**16**	118	28	66	40
**17**	2	0	95	57
**18**	1	0	112	66
**19**	1	0	108	69

(a) Raw counts.

ℓ	AICO Regression 5%	1%	AICO Classification 5%	1%
**1**	2000	2000	2000	2000
**2**	2000	2000	2000	2000
**3**	2000	2000	1872	1872
**4**	2000	2000	1849	1847
**5**	2000	2000	1845	1844
**6**	2000	2000	2000	2000
**7**	2000	2000	2000	2000
**8**	2000	2000	2000	2000
**9**	2000	2000	2000	2000
**10**	2000	2000	2000	2000
**11**	2000	2000	2000	2000
**12**	2000	2000	2000	2000
**13**	0	0	12	11
**14**	0	0	22	13
**15**	8	1	27	19
**16**	14	3	35	30
**17**	0	0	43	30
**18**	0	0	46	29
**19**	0	0	51	34

(b) Bonferroni-adjusted at $\alpha/19$.

Table S5. Regression and classification AICO test results for $2{,}000$ independent experiments at two standard significance levels ($\alpha = 5\%$ and $\alpha = 1\%$). We report the total number of rejections (out of $2{,}000$) per feature ℓ. Panel (a) shows the raw counts at nominal α; panel (b) applies a Bonferroni adjustment with per-feature threshold $\alpha/19$. The training set size is 10^6 and the test set size is $N = 5 \cdot 10^5$.

classification task (Appendix S2.1), using $M_0 = 0$.

The AICO test concerns a model-relative null. In the data-generating process (DGP), a feature is null when the response does not depend on it; in our design these are features 13 through 19. For the AICO test, by contrast, a feature is null when the feature-effect distribution induced by the fitted model and masking specification has median $M^\ell \leq 0$, so that masking the feature does not systematically degrade predictive performance. The two notions need not coincide. Trained on finitely many samples, the fitted model may attach a small positive effect to a DGP-null feature. Moreover, because the feature effect depends on the masking specification, a feature that is null in the DGP need not be AICO-null under a given masking scheme, even when the fitted model accurately represents the underlying response mechanism. In either case, the resulting feature-effect distribution may satisfy $M^\ell > 0$. A rejection is then a false discovery relative to the DGP null, but not relative to the AICO null. Since the size property applies only when $M^\ell \leq 0$, we restrict the demonstration to fitted models for which this condition holds.

For each DGP-null feature ℓ, we repeatedly draw a training set D_1 of 10^6 samples, fit f as in Section 4 and Appendix S2.1, and estimate M^ℓ on a fresh sample of 10^8 points, which is sufficiently large to accurately estimate the sign of M^ℓ. Separately for each feature, we retain the first 100 fitted models for which the estimated median satisfies $M^\ell \leq 0$; this retained set generally differs across features. For each retained model, we estimate its rejection probability by applying the AICO test to 10,000 independent test sets of size $N = 5 \cdot 10^5$ and recording the fraction of rejections. Masking uses the same reference-value specification as in the main experiments. For each feature, Table S8 reports the mean, standard deviation, and maximum of these estimated rejection probabilities across the retained models, together with the number of estimates exceeding α.

The results are consistent with the finite-sample size property in both settings. For every feature, the mean estimated rejection probability is at or below α, and it is far smaller for fitted models whose estimated median effects are well below zero. The few estimates that exceed α occur for fitted models whose estimated median effects are close to the boundary $M^\ell = 0$. At this boundary, the rejection probability is equal to or close to α, so finite Monte Carlo estimation can produce rejection-frequency estimates slightly above α. Increasing the number of repeated test sets reduces the magnitude of these exceedances but need not eliminate their occurrence. No fitted model whose estimated median effect is well below zero produces a rejection-frequency estimate above the nominal level, consistent with the exact finite-sample validity of the test.

The relatively large number of exceedances for regression features 15 and 16 may also be related to their correlation in the data-generating process. Correlation can make it more difficult for the fitted model to distinguish among features, potentially leading to estimated median feature effects that lie closer to the boundary $M^\ell = 0$ and thereby increasing the frequency of Monte Carlo exceedances.

	AICO			
	Regression		Classification	
ℓ	5%	1%	5%	1%
**1**	2000	2000	2000	2000
**2**	2000	2000	2000	1999
**3**	2000	2000	2000	2000
**4**	2000	2000	1996	1996
**5**	2000	2000	1999	1998
**6**	2000	2000	2000	2000
**7**	2000	2000	2000	2000
**8**	2000	2000	2000	2000
**9**	2000	2000	2000	2000
**10**	2000	2000	2000	2000
**11**	2000	2000	2000	2000
**12**	2000	2000	2000	2000
**13**	3	0	5	1
**14**	1	0	5	1
**15**	0	0	2	1
**16**	1	0	5	1
**17**	1	0	15	10
**18**	0	0	14	9
**19**	0	0	14	8

(a) Raw counts.

	AICO			
	Regression		Classification	
ℓ	5%	1%	5%	1%
**1**	2000	2000	2000	2000
**2**	2000	2000	1999	1997
**3**	2000	2000	2000	2000
**4**	2000	2000	1996	1996
**5**	2000	2000	1998	1998
**6**	2000	2000	2000	2000
**7**	2000	2000	2000	2000
**8**	2000	2000	2000	2000
**9**	2000	2000	2000	2000
**10**	2000	2000	2000	2000
**11**	2000	2000	2000	2000
**12**	2000	2000	2000	2000
**13**	0	0	0	0
**14**	0	0	0	0
**15**	0	0	1	1
**16**	0	0	0	0
**17**	0	0	9	5
**18**	0	0	6	3
**19**	0	0	3	3

(b) Bonferroni-adjusted at $\alpha/19$.

Table S6. Regression and classification model AICO test results for $2{,}000$ independent training/testing experiments at two standard significance levels ($\alpha = 5\%$ and $\alpha = 1\%$). The model is a best-three-of-five ensemble, and masking uses the OLS-estimated conditional-mean reference value. We report the total number of rejections (out of $2{,}000$) per feature ℓ. Panel (a) shows the raw counts at nominal α; panel (b) applies a Bonferroni adjustment with per-feature threshold $\alpha/19$. The training set size is 10^6 and the test set size is $N = 5 \cdot 10^5$.

S3. Multiple data splits: Cross-fitting

Data splitting into training and testing sets (see Section 1) can reduce statistical efficiency and testing power. These issues can be mitigated by using multiple data splits, so that each sample is used for both training and testing. Our testing procedure extends naturally to this cross-fitting setting.

Consider a partition of the data $\{Z_i\}$ into $K \geq 2$ disjoint folds of approximately equal size. For each $k \in [K]$, the folds excluding k form the training set I_{1k} used to construct the model f_k, while fold k serves as the test set I_{2k}. Based on I_{1k} and f_k, we construct fold-specific feature effects $\Delta_k^\ell(Z_i)$ for $i \in I_{2k}$. Several approaches can then be used to implement the testing procedure and construct a p-value for feature ℓ; see (3–5), among others.

One approach is to perform fold-specific tests on $\{\Delta_k^\ell(Z_i)\}_{i \in I_{2k}}$ for each $k \in [K]$, yielding p-values $p_1, \dots, p_K$. A simple aggregation rule rejects the overall null if at least one fold-specific null is rejected, with corresponding p-value $\min(p_1, \dots, p_K)$ or, with Bonferroni correction, $\min(K \cdot \min(p_1, \dots, p_K), 1)$. These aggregated p-values inherit the validity of the fold-specific p-values, and their conditional distributions given the fold-specific test statistics follow from the uniform law (11).

An alternative approach applies the testing procedure to the pooled set of feature effects $\bigcup_{k=1}^K \{\Delta_k^\ell(Z_i) : i \in I_{2k}\}$, yielding a single decision and p-value following Section 2. The theoretical validity of this approach is unclear, since the $\Delta_k^\ell(Z_i)$ may be dependent across folds due to overlapping training samples.

As the number of folds K increases, both approaches fit the model in each fold using a larger fraction $(1 - 1/K)$ of the data, which can improve the quality of the fitted model—and hence the power of the test—by more faithfully capturing subtle feature-response relationships. Cross-fitting also mitigates "split lottery" effects, wherein atypical train-test partitions that are unrepresentative of the underlying population can arise by chance, thereby reducing variability across random train-test partitions. These benefits must be balanced against additional computational cost as K grows. Moreover, under the first aggregation approach, larger K also decreases the per-fold testing sample size and tightens the Bonferroni correction, which can reduce power. Tansey et al. (3) suggest that in practice $K = 5$ folds are sufficient to attain high power for both aggregation approaches.

S3.1. Numerical results. We re-run the regression and classification experiments of Section 4 and Appendix S2.1 in a cross-fitting setting with $K = 5$ folds on the full sample of $1.5 \cdot 10^6$ observations. In each fold, the neural network model and reference values are constructed using the corresponding training partition, and the resulting feature effects are evaluated on the held-out fold, as previously described. All remaining configurations of the experiment—including the data-generating process, network

ℓ	AICO		CPT		HRT		LOCO	
	5%	1%	5%	1%	5%	1%	5%	1%
**1**	1000	1000	1000	1000	1000	1000	1000	1000
**2**	1000	1000	1000	1000	1000	1000	1000	1000
**3**	1000	1000	1000	1000	1000	1000	1000	1000
**4**	1000	1000	1000	1000	1000	1000	1000	1000
**5**	1000	1000	1000	1000	1000	1000	1000	1000
**6**	1000	1000	1000	1000	1000	1000	1000	1000
**7**	1000	1000	1000	1000	1000	1000	1000	1000
**8**	1000	1000	1000	1000	1000	1000	1000	1000
**9**	1000	1000	1000	1000	1000	1000	1000	1000
**10**	1000	1000	1000	1000	1000	1000	1000	1000
**11**	1000	1000	1000	1000	1000	1000	1000	1000
**12**	1000	1000	1000	1000	1000	1000	1000	1000
**13**	7	1	59	14	59	14	98	49
**14**	3	2	42	13	41	9	97	48
**15**	49	10	53	14	45	8	118	64
**16**	45	9	74	17	60	13	125	62
**17**	5	1	76	19	49	9	101	59
**18**	3	1	68	10	55	13	90	44
**19**	8	2	77	13	46	13	95	48

Table S7. Regression model test results for 1,000 independent training/testing experiments at two standard significance levels ($\alpha = 5\%$ and $\alpha = 1\%$). The table compares AICO, CPT, HRT, and LOCO procedures, showing the total number of rejections across 1,000 trials. The training set size is 10^4 and the test set size is $N = 5 \cdot 10^3$.

architecture and training procedure, and feature effect specification—are identical to those used in Section 4.

Table S9 reports results under the two aggregation schemes for ten independent trials at the 5% and 1% levels. The results are similar to the single train-test split results reported in the first two columns of Tables 1 and S1. Across all trials, features entering the DGP ($\ell = 1, \ldots, 12$) are identified as significant under both aggregation schemes for both regression and classification. Features that are null in the DGP are identified as significant only occasionally. This modest increase in such identifications could be due to fold-to-fold estimation variability: fitting K fold-specific models can occasionally yield suboptimal fits, and the resulting noise may propagate into the computed feature effects. Increasing K enlarges the training fraction $(1 - 1/K)$ within each fold and can mitigate such suboptimal-fit events, although for the first aggregation approach this must be weighed against smaller per-fold test sets and a tighter Bonferroni aggregation. Overall, the results are similar across the two aggregation schemes, and neither exhibits a clear advantage in these experiments.

S4. Panel data

This appendix explains how panel data, which may exhibit cross-sectional or time-series dependence, can be handled in AICO. Consider panel data $\{Z_{i,t}\}$, where $Z_{i,t} = (X_{i,t}, Y_{i,t})$, with $i = 1, \ldots, m$ indexing units and $t = 1, \ldots, T$ indexing time. We partition the index set $\{(i,t) : i \in [m], t \in [T]\}$ into two disjoint subsets, I_1 and I_2, such that each pair belongs to exactly one subset. This setup accommodates common data-splitting schemes, including temporal splits (distinguishing between in-sample and out-of-sample time periods), unit-based splits (assigning each unit's entire time series to either I_1 or I_2), or combinations thereof. For an unbalanced panel, in which units may be observed at different times, only observed pairs (i,t) are assigned to I_1 or I_2.

S4.1. Conditioning. Conditioning entails focusing on a suitable subset $I \subseteq I_2$ when constructing the feature effect. If some samples in I_2 are dependent, we can restrict attention to the subset I of independent samples $(i,t) \in I_2$. This reduces the test set size but helps avoid inference distortions due to unaccounted dependence, see (6), for example. For instance, in the presence of temporal dependence, one possible choice is to construct I by restricting attention to samples at a given time t, yielding the subset $I = \{(\cdot, t) \in I_2\}$ and corresponding feature effects $\{\Delta^\ell(Z_{i,t}) : (i,t) \in I\}$. This approach yields a sequence of tests and a corresponding time series of p-values, importance scores, and confidence intervals, whose evolution over time may provide additional insight.

For constructing masking reference values using the training data (see Section 1.2), the training set I_1 is treated as a collection of $|I_1|$ samples—with unit and time information tied to each—fully exploiting the richness of the panel structure. For each $(i,t) \in I$, the masking reference values can draw information not only from all training samples I_1 but also from testing samples corresponding to the same unit but not in the considered subset, $\{X_{i,\cdot} : (i, \cdot) \in I_2 \setminus I\}$. This flexibility enables

ℓ	AICO															
	Regression								Classification							
	$\alpha = 5\%$				$\alpha = 1\%$				$\alpha = 5\%$				$\alpha = 1\%$			
	Mean	SD	Max	#Exceed	Mean	SD	Max	#Exceed	Mean	SD	Max	#Exceed	Mean	SD	Max	#Exceed
**13**	0.0003	0.0002	0.0011	0	0.0000	0.0000	0.0002	0	0.0046	0.0081	0.0469	0	0.0007	0.0015	0.0105	1
**14**	0.0003	0.0002	0.0010	0	0.0000	0.0001	0.0004	0	0.0047	0.0081	0.0439	0	0.0006	0.0014	0.0081	0
**15**	0.0305	0.0138	0.0629	9	0.0055	0.0032	0.0146	9	0.0031	0.0071	0.0430	0	0.0004	0.0012	0.0082	0
**16**	0.0322	0.0134	0.0613	9	0.0059	0.0030	0.0127	10	0.0049	0.0089	0.0478	0	0.0008	0.0018	0.0107	1
**17**	0.0003	0.0002	0.0010	0	0.0000	0.0000	0.0002	0	0.0041	0.0069	0.0422	0	0.0005	0.0012	0.0082	0
**18**	0.0002	0.0002	0.0007	0	0.0000	0.0000	0.0002	0	0.0060	0.0112	0.0553	1	0.0009	0.0020	0.0107	1
**19**	0.0003	0.0002	0.0009	0	0.0000	0.0000	0.0002	0	0.0053	0.0093	0.0383	0	0.0008	0.0018	0.0090	0

Table S8. Numerical demonstration of the finite-sample validity of the AICO test. For each DGP-null feature ℓ, among the 100 fitted models retained for that feature because their estimated median effect satisfies $\hat{M}^\ell \leq 0$, we report the mean, standard deviation, and maximum of the estimated rejection probability, each estimated over $10{,}000$ independent test sets, together with the number of retained models whose estimated rejection probability exceeds α (#Exceed). The training set size is 10^6 and the test set size is $N = 5 \cdot 10^5$.

unit-specific and time-dependent reference values, such as lagged feature values (e.g., the previous period's feature value) or historical aggregations, thereby enhancing the contextual relevance of the reference value.

Conditioning is applicable beyond temporal dependence. For example, under spatial dependence, one may restrict attention to at most one observation per region or neighborhood to promote approximate independence, although the additional randomness induced by subsampling should be taken into account when interpreting the results.

S4.2. Grouping. An alternative grouping approach enables joint testing at the unit level, across time-series observations. The idea is to aggregate feature effects within a unit i so that any time-series dependence is contained within groups. See (7) for feature importance analysis in this context and (8), among others, for analysis of related approaches in a regression setting.

We treat the testing set I_2 as a collection of $|I_2|$ samples and compute the masked and unmasked predictions $f(m^\ell(X_{i,t}))$ and $f(u^\ell(X_{i,t}))$ for $(i,t) \in I_2$, where $m^\ell(X_{i,t})$ and $u^\ell(X_{i,t})$ are masked and unmasked feature vectors constructed as in Section 1. We group the predictions and corresponding true responses by unit i to obtain the unit-level feature effect

$$L^*(g_i^\ell, Y_i^*) - L^*(h_i^\ell, Y_i^*), \tag{3}$$

where L^* is a trajectory-level loss function, $g_i^\ell = \{f(m^\ell(X_{i,\cdot})) : (i,\cdot) \in I_2\}$, $h_i^\ell = \{f(u^\ell(X_{i,\cdot})) : (i,\cdot) \in I_2\}$, and $Y_i^* = \{Y_{i,\cdot} : (i,\cdot) \in I_2\}$. Unlike the setting of Section 1, where the loss function L evaluates one prediction against one true response, the trajectory-level loss function L^* evaluates the entire sequence of predictions against the corresponding sequence of true responses.

Trajectory-level loss functions can extend familiar loss metrics. For instance, one may use the *mean* squared error, averaging squared errors across time steps. More complex specifications can exploit richer temporal structure, such as correlation (Information Coefficient, IC), the error in maximum drawdown, or other time-series evaluations tailored to specific domains.

More broadly, the grouping approach extends beyond temporal dependence structures to cross-sectional and spatial dependence structures. For spatially dependent data, one may group testing samples by region or neighborhood, under the assumption that neighborhoods are approximately independent. Likewise, for repeated-measures data, grouping by the underlying entity or experimental context can provide an effective way to account for within-group dependence while preserving the assumptions needed for inference across groups.

S4.3. Hybrid approach. Conditioning and grouping can be combined. For instance, suppose we wish to analyze a feature's impact on model performance over an extended period. A longer period helps smooth short-term noise but makes single-period conditioning insufficient. Conversely, a shorter period preserves data relevance but may preclude grouping across testing time horizons. This limitation can be addressed by first conditioning on a time frame of suitable length and then grouping the conditioned data by units corresponding to each time–unit pair. As with conditioning alone, this procedure can be repeated across overlapping time windows, yielding a rolling-window analysis with smoothed single-period results.

Another notable use case of the hybrid method is assessing per-unit consistency of feature performance—how consistent over time is a feature's impact on model behavior for a given unit? A longer window mitigates short-term fluctuations, and when the multi-period loss function aggregates single-period losses, it also captures an additional dimension of per-unit consistency.

For example, consider a multi-period loss function L^* defined as the average of a single-period function, such as the mean squared error. In this case, the unit-level feature effect Eq. (3) is effectively the average of the unit–time-level effects $\Delta^\ell(Z_{i,t})$ and thus reflects a degree of consistency over time. The longer the time window, the stronger the emphasis on consistency over time, as a higher bar is set for variation in feature impact.

ℓ	Regression				Classification			
	AICO		Pooled AICO		AICO		Pooled AICO	
	5%	1%	5%	1%	5%	1%	5%	1%
**1**	10	10	10	10	10	10	10	10
**2**	10	10	10	10	10	10	10	10
**3**	10	10	10	10	10	10	10	10
**4**	10	10	10	10	10	10	10	10
**5**	10	10	10	10	10	10	10	10
**6**	10	10	10	10	10	10	10	10
**7**	10	10	10	10	10	10	10	10
**8**	10	10	10	10	10	10	10	10
**9**	10	10	10	10	10	10	10	10
**10**	10	10	10	10	10	10	10	10
**11**	10	10	10	10	10	10	10	10
**12**	10	10	10	10	10	10	10	10
**13**	0	0	0	0	0	0	0	0
**14**	0	0	0	0	1	1	0	0
**15**	0	0	1	0	1	1	0	0
**16**	0	0	3	1	1	1	0	0
**17**	0	0	0	0	1	1	0	0
**18**	0	0	0	0	4	4	3	2
**19**	0	0	0	0	2	2	2	1

Table S9. AICO test results for 5-fold cross-fitting for the regression and classification tasks of Section 4 and Appendix S2.1, with a dataset of size $1.5 \cdot 10^6$. The entries report the number of rejections (out of 10) for each feature $\ell \in [d]$ at levels $\alpha = 5\%$ and $\alpha = 1\%$. The column "AICO" reports results obtained under the scheme that aggregates separate fold-specific test results (p-values). The column "Pooled AICO" reports results obtained under the scheme that entails pooling of fold-specific feature effects.

S5. Missing data

In practice, many datasets contain missing feature values, which are handled via a variety of deletion or imputation schemes. In general, when samples with missing or imputed feature values can be identified,[†] we recommend excluding them from the test set in a feature-wise manner: when testing feature ℓ, we drop only samples i for which X_i^ℓ is missing—regardless of the missingness of other features—to preserve sample size as much as possible. This approach avoids ambiguity in the unmasking operation: $u^\ell(\cdot)$ is intended to reintroduce the information in feature ℓ into the model input, which is ill-defined when the true value X_i^ℓ is missing. Moreover, this feature-wise exclusion reduces potential distortion in the feature effects due to overlap between imputed values and masking reference values. Finally, feature-wise exclusion reduces the extent to which AICO results depend on the chosen imputation scheme, due to the possibility that different imputations can yield different inferences.

Under Missing Completely At Random (MCAR), wherein missingness is independent of the data, our approach does not systematically bias the resulting inference. While it may decrease power and increase variance due to a reduced effective test sample size, our approach can drop substantially fewer samples than common strategies such as listwise deletion, wherein any sample with at least one missing feature value is removed. When missingness depends on the data—e.g., under Missing At Random (MAR) or Missing Not At Random (MNAR)—excluding samples with missing X_i^ℓ can change the target estimand. Following (10), the resulting inference can still be interpreted as "observed-data feature importance", which quantifies importance under the observed-data distribution. In some settings, this estimand may be more relevant than "full-data feature importance"—defined under the hypothetical distribution with perfect observation—for example, during model development when the missingness pattern in the retrospective dataset is expected to persist after deployment.

Multiple imputation (MI) and inverse-probability weighting (IPW) are also widely used for handling missing data in estimation problems (10–12). For example, Williamson and Huang (13) combine MI with an algorithm-agnostic variable-importance measure to enable variable selection under missingness with flexible learning algorithms beyond finite-dimensional statistical models. MI can also be incorporated into AICO by running AICO on each imputed dataset and then pooling inferences for the median feature-effect estimate across imputations using Rubin's rules, leveraging the asymptotic normality of the sample median (via the Central Limit Theorem for medians). IPW can also be incorporated into AICO by reweighting the feature effects, but this would require generalizing the sign test to a weighted setting (14–17), which we leave for future work.

While more sophisticated imputation can outperform simple deletion in some settings, several studies suggest this is not guaranteed in general. For example, Seijo-Pardo et al. (18) emphasize that constant-value imputations (e.g., mean imputation) can distort covariance structure and thereby bias downstream feature-importance estimates and rankings, whereas complete-case

[†] A common strategy is to augment the input vector with missingness-indicator features to distinguish imputed from observed values during model training (see, for example, (9)).

AICO, Conditional on MARRIAGE = Single, Level $\alpha = 1\%$											
				p-value Distribution		Randomized One-Sided CI			Non-Randomized Two-Sided CI		
Rank	Variable	Median	P(reject)	Lower	Upper	83.0%	17.0%	Coverage	Lower	Upper	Coverage
1	PAY_0	0.151	1.000	1.833e-248	5.763e-248	0.143	0.143	0.99	0.142	0.158	0.99023
2	PAY_2	0.130	1.000	0.000	0.000	0.125	0.125	0.99	0.124	0.136	0.99023
3	PAY_3	0.100	1.000	1.072e-295	3.822e-295	0.095	0.095	0.99	0.095	0.105	0.99023
4	PAY_4	0.063	1.000	1.402e-260	4.555e-260	0.056	0.057	0.99	0.056	0.069	0.99023
5	PAY_5	0.040	1.000	4.099e-183	1.071e-182	0.037	0.037	0.99	0.037	0.044	0.99023
6	PAY_6	0.029	1.000	3.112e-171	7.848e-171	0.028	0.028	0.99	0.027	0.031	0.99023
7	AGE	0.002	1.000	2.601e-48	4.147e-48	0.002	0.002	0.99	0.002	0.002	0.99023
8	SEX	0.001	1.000	1.972e-16	2.560e-16	0.001	0.001	0.99	0.001	0.002	0.99023
-	LIMIT_BAL	-4.292e-04	0.000	1.000	1.000	-0.002	-0.002	0.99	-0.002	0.000	0.99511
-	BILL_AMT1	-0.005	0.000	1.000	1.000	-0.007	-0.007	0.99	-0.007	-0.003	0.99023
-	BILL_AMT2	-0.001	0.000	1.000	1.000	-0.002	-0.002	0.99	-0.002	-8.918e-04	0.99023
-	BILL_AMT3	4.366e-05	0.000	0.376	0.388	-1.597e-05	-1.038e-05	0.99	-3.777e-05	2.814e-04	0.99023
-	BILL_AMT4	-4.339e-04	0.000	1.000	1.000	-6.109e-04	-6.105e-04	0.99	-6.237e-04	-2.882e-04	0.99023
-	BILL_AMT5	-1.737e-04	0.000	1.000	1.000	-3.338e-04	-3.327e-04	0.99	-3.612e-04	-3.330e-05	0.99023
-	BILL_AMT6	-1.147e-04	0.000	1.000	1.000	-2.326e-04	-2.315e-04	0.99	-2.509e-04	0.000	0.99474
-	PAY_AMT1	-0.014	0.000	1.000	1.000	-0.015	-0.015	0.99	-0.016	-0.011	0.99023
-	PAY_AMT2	-0.010	0.000	1.000	1.000	-0.012	-0.012	0.99	-0.012	-0.009	0.99023
-	PAY_AMT3	-0.006	0.000	1.000	1.000	-0.008	-0.008	0.99	-0.008	-0.005	0.99023
-	PAY_AMT4	-4.392e-04	0.000	1.000	1.000	-7.181e-04	-7.118e-04	0.99	-7.488e-04	-1.825e-04	0.99023
-	PAY_AMT5	-0.002	0.000	1.000	1.000	-0.003	-0.003	0.99	-0.003	-0.002	0.99023
-	PAY_AMT6	-0.002	0.000	1.000	1.000	-0.002	-0.002	0.99	-0.002	-0.001	0.99023
-	EDUCATION	0.001	0.000	0.012	0.013	-7.817e-05	-4.987e-05	0.99	-2.034e-04	0.002	0.99023

Table S10. AICO test summary for the credit card default dataset. The test is conditioned on the samples for which `MARRIAGE` = Single. Percentage heads: probabilities of the two possible lower endpoints of the randomized CI.

analysis avoids such distortions by relying only on observed data. Additionally, the broader machine learning survey of (19) notes that deletion-based methods are commonly used in practice due to their simplicity, low computational cost, and minimal modeling assumptions, despite the reduction in sample size. These considerations are particularly relevant for AICO, where computational efficiency and minimal assumptions are central.

Note that when applying the grouping approach in Appendix S4.2, the exclusion can be performed at the prediction level—i.e., on $f(m^\ell(X_{i,t}))$, $f(u^\ell(X_{i,t}))$, and $Y_{i,t}$—rather than at the feature-effect level Eq. (3), to preserve sample size. For example, in a panel setting, if unit i has a missing value only at a single time step t (i.e., $X^\ell_{i,t}$ is missing, while $X^\ell_{i,s}$ is observed for all $s \neq t$), it may suffice to drop only the corresponding predictions $f(m^\ell(X_{i,t}))$ and $f(u^\ell(X_{i,t}))$ and the associated response $Y_{i,t}$ from unit i's grouped trajectory. In other words, when only one point is missing in the time series, we drop that time point rather than the entire trajectory.

Finally, each AICO test is conditional on the fitted model f, so any bias in or limitations of the fitted model can carry over to the test and feature-importance results. Accordingly, missing data and the method used to handle it in the training set affect AICO primarily through model fitting. Van Ness et al. (9) show that the Missing Indicator Method coupled with mean imputation, as adopted in our empirical experiments in Section 5, is a strong approach while being substantially more efficient than more complicated imputation schemes.

S6. Additional results for the credit dataset

We further explore the role of individual features by running the test on subsets of the test set. For instance, Table S10 reports results for the 4,029 (out of 7,500) samples for which `MARRIAGE` = Single. While the test does not reject the null for `EDUCATION` at the 1% level, it rejects at the 5% level, as the distribution of its p-value is concentrated below 0.05. When the test is restricted to samples with `MARRIAGE` = Married, the null for `EDUCATION` is no longer rejected. These findings provide evidence that educational level contributes predictive information for unmarried borrowers, but provide no comparable evidence for married borrowers.

References

1. E Lehmann, J Romano, *Testing Statistical Hypotheses.* (Springer), (2005).

2. CJ Geyer, GD Meeden, Fuzzy and randomized confidence intervals and p-values. *Stat. Sci.* **20**, 358–366 (2005).
3. W Tansey, V Veitch, H Zhang, R Rabadan, DM Blei, The holdout randomization test for feature selection in black box models. *J. Comput. Graph. Stat.* **31**, 151–162 (2022).
4. M Liu, E Katsevich, L Janson, A Ramdas, Fast and powerful conditional randomization testing via distillation. *Biometrika* **109**, 277–293 (2022).
5. FR Guo, RD Shah, Rank-transformed subsampling: inference for multiple data splitting and exchangeable p-values. *J. Royal Stat. Soc. Ser. B: Stat. Methodol.* **87**, 256–286 (2025).
6. AC Cameron, DL Miller, A practitioner's guide to cluster-robust inference. *J. Hum. Resour.* **50**, 317–372 (2015).
7. BD Williamson, EE Moodie, GE Simon, RC Rossom, SM Shortreed, Inference on summaries of a model-agnostic longitudinal variable importance trajectory with application to suicide prevention. *Annals Appl. Stat.* **20**, 1340–1363 (2026).
8. KY Liang, S Zeger, Longitudinal data analysis using generalized linear models. *Biometrika* **73**, 13–22 (1986).
9. M Van Ness, TM Bosschieter, R Halpin-Gregorio, M Udell, The missing indicator method: From low to high dimensions in *Proceedings of the 29th ACM SIGKDD Conference on Knowledge Discovery and Data Mining.* pp. 5004–5015 (2023).
10. H Von Kleist, J Wendland, I Shpitser, C Marr, Feature importance metrics in the presence of missing data in *Forty-second International Conference on Machine Learning, ICML.* (2025).
11. BD Williamson, et al., Assessing treatment effects in observational data with missing confounders: A comparative study of practical doubly-robust and traditional missing data methods. *Stat. Medicine* **45**, e70366 (2026).
12. SR Seaman, IR White, Review of inverse probability weighting for dealing with missing data. *Stat. Methods Med. Res.* **22**, 278–295 (2013).
13. BD Williamson, Y Huang, Flexible variable selection in the presence of missing data. *The Int. J. Biostat.* **20**, 347–359 (2024).
14. J McDonald, PD Gerard, CS McMahan, WR Schucany, Exact-permutation-based sign tests for clustered binary data via weighted and unweighted test statistics. *J. Agric. Biol. Environ. Stat.* **21**, 698–712 (2016).
15. C Ahn, F Hu, SC Lee, Relative efficiency of unequal versus equal cluster sizes for the nonparametric weighted sign test estimators in clustered binary data. *Drug Inf. J.* **46**, 428–433 (2012).
16. D Larocque, J Nevalainen, H Oja, A weighted multivariate sign test for cluster-correlated data. *Biometrika* **94**, 267–283 (2007).
17. SA Kassam, JB Thomas, Nonparametric weighted-signs tests for location. *SIAM J. on Appl. Math.* **32**, 649–652 (1977).
18. B Seijo-Pardo, et al., Analysis of imputation bias for feature selection with missing data in *European Symposium on Artificial Neural Networks, Computational Intelligence and Machine Learning, ESANN.* (2018).
19. T Emmanuel, et al., A survey on missing data in machine learning. *J. Big Data* **8**, 140 (2021).
